# Mixed-valent, heteroleptic homometallic diketonates as templates for the design of volatile heterometallic precursors†
†Electronic supplementary information (ESI) available: Experimental procedures, synthetic details, IR spectra, X-ray powder diffraction patterns, computational details, TEM images and phase analysis of thermal decomposition products. CCDC 1040548–1040553. For ESI and crystallographic data in CIF or other electronic format see DOI: 10.1039/c4sc04002c



**DOI:** 10.1039/c4sc04002c

**Published:** 2015-02-25

**Authors:** Craig M. Lieberman, Alexander S. Filatov, Zheng Wei, Andrey Yu. Rogachev, Artem M. Abakumov, Evgeny V. Dikarev

**Affiliations:** a Department of Chemistry , University at Albany , Albany , NY 12222 , USA . Email: edikarev@albany.edu ; Fax: +1-518-442-3462 ; Tel: +1-518-442-4401; b Department of Biological and Chemical Sciences , Illinois Institute of Technology , Chicago , IL 60616 , USA; c EMAT , University of Antwerp , Groenenborgerlaan 171 , B-2020 Antwerp , Belgium

## Abstract

A novel series of mixed-valent, heteroleptic transition metal diketonates that can be utilized as prospective single-source precursors for the low-temperature preparation of oxide materials are reported. The first mixed-valent iron β-diketonates with different Fe^III^/Fe^II^ ratios have been synthesized by applying the mixed-ligand approach. Based on nearly quantitative reaction yields and analysis of iron–oxygen bonds, these compounds were formulated as [Fe^III^(acac)_3_][Fe^II^(hfac)_2_] (**1**) and [Fe^II^(hfac)_2_][Fe^III^(acac)_3_][Fe^II^(hfac)_2_] (**2**). In the above heteroleptic complexes, the Lewis acidic, coordinatively unsaturated Fe^II^ centers chelated by two hfac (hexafluoroacetylacetonate) ligands with electron-withdrawing substituents maintain bridging interactions with oxygen atoms of electron-donating acac (acetylacetonate) groups that chelate the neighboring Fe^III^ atoms. Switching the ligands on Fe^III^ and Fe^II^ atoms in starting reagents resulted in the instant ligand exchange between iron centers and in yet another polynuclear homometallic diketonate [Fe^II^(hfac)_2_][Fe^III^(acac)_2_(hfac)][Fe^II^(hfac)_2_] (**3**) that adheres to the same bonding pattern as in complexes **1** and **2**. The proposed synthetic methodology has been extended to design heterometallic diketonates with different M : M′ ratios. Homometallic parent molecules have been used as templates to obtain heterometallic mixed-valent [Fe^III^(acac)_3_][Mn^II^(hfac)_2_] (**4**) and [Ni^II^(hfac)_2_][Fe^III^(acac)_3_][Ni^II^(hfac)_2_] (**5**) complexes. The combination of two different diketonate ligands with electron-donating and electron-withdrawing substituents was found to be crucial for maintaining the above mixed-valent heterometallic assemblies. Theoretical investigation of two possible “isomers”, [Fe^III^(acac)_3_][Mn^II^(hfac)_2_] (**4**) and [Mn^III^(acac)_3_][Fe^II^(hfac)_2_] (**4′**) provided an additional support for the metal site assignment giving a preference of 9.78 kcal mol^–1^ for the molecule **4**. Heterometallic complexes obtained in the course of this study have been found to act as effective single-source precursors for the synthesis of mixed-transition metal oxide materials M_*x*_M′2–*x*O_3_ and M_*x*_M′1–*x*O. The title highly volatile precursors can be used for the low-temperature preparation of both amorphous and crystalline heterometallic oxides in the form of thin films or nanosized particles that are known to operate as efficient catalysts in oxygen evolution reaction.

## Abstract 2

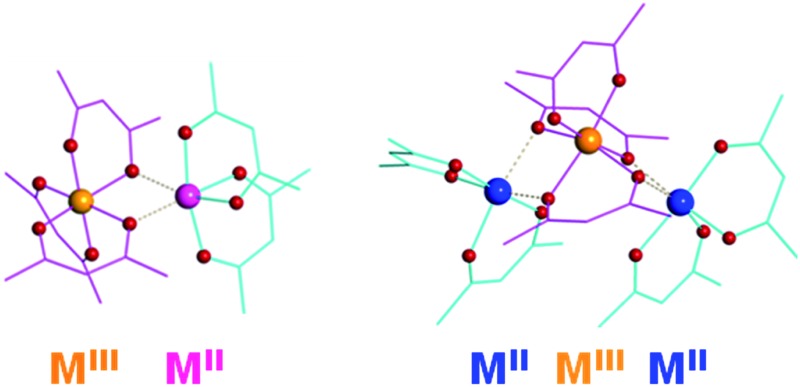
A unique series of mixed-valent transition metal complexes (M^III^ = Fe; M^II^ = Fe, Mn, Ni) have been designed using a combination of diketonate ligands with electron-withdrawing (blue) and electron-donating (pink) substituents.

## Introduction

One of the greatest technological challenges facing our global well-being is the development of renewable energy sources. Over the past thirty years, a great deal of research has been focused on the development of new catalysts for the oxygen evolution reaction (OER).^1^ The so-called water splitting to hydrogen and oxygen is vital for the advancement towards clean and sustainable energy storage in the form of chemical fuels.^2^ The development of new and cost-effective catalysts represents a key challenge, since the electrolysis of water requires voltages in substantial excess of the thermodynamic potential for its oxidation.^3^ Currently, the most active catalysts for the oxygen evolution reaction are based on precious metals such as Ru and Ir.^4^ Due to the high cost and poor long-term stability of these materials in alkaline solutions, their widespread commercial utilization will be impractical.^5^ Various alternatives have been thoroughly studied including both pure and mixed transition metal oxides,^6^ perovskites incorporating late first-row transition metal ions,^7^ and spinels.^8^ Among these options, mixed-transition metal oxides appear to be the most promising substitutes for the catalysts based on precious metals. Thus, heterometallic Fe–Ni oxide thin films grown by electrodeposition technique reveal far greater oxygen evolution reactivity than the corresponding parent homometallic materials.^9^ Furthermore, Fe–Ni oxides have been shown^10^ to exhibit catalytic activities comparable to those of IrO_2_ and RuO_2_. More recently, the preparation of amorphous mixed-metal oxides as proficient catalysts for the oxygen evolution reaction has been developed.^11^ Through the use of a photochemical metal-organic deposition (PMOD) technique, various amorphous transition metal oxide films have been assembled. The presence of moderate amounts of iron (20–40%) was shown^12^ to improve the catalytic activity in nickel oxide-based films. Similar behavior was also observed^13^ in amorphous Fe–Ni oxide films prepared by electrodeposition methods, with optimal catalytic performance displayed by materials with 1 : 2 and 1 : 2.6 Fe : Ni ratios, even though the metal content in the active catalysts differs from the stoichiometry of the precursor solution employed.

Our research is focused on the preparation of heterometallic β-diketonate single-source precursors (SSPs) and their application for the low-temperature synthesis of mixed-metal oxides.^14^ Until now, the employment of metal β-diketonates as precursors for OER catalysts was relatively unexplored and limited by a few examples involving homometallic Ir^III^ acetylacetonate.^15^ At the same time, the use of metal β-diketonates as MOCVD precursors for oxide materials is attractive due to high volatility of these compounds and their rigid structures that ensure better control over the precursor composition.^16^ The application of SSPs helps to overcome incompatibilities in behavior of multiple precursors as well as to avoid the high-temperature/pressure synthetic conditions characteristic of conventional solid state routes.^17^ Specifically, we design precursors that are volatile, soluble in common solvents, and have discrete molecular structures to ensure that the heterometallic core is retained in the gas phase or in solution. Such precursors can be effectively utilized for the low-temperature preparation of both amorphous and highly crystalline mixed-metal oxides in the form of thin films or nanosized particles. The morphology of the target oxide materials is quite important as it can bring about fundamental modifications of underlying properties.^18^ The larger surface area provided by nanoparticles is believed to enhance the catalytic performance of metal oxides.^19^ In order to make the precursors appealing for commercial applications, their synthesis should be optimized as a simple, high-yield reaction procedure that is run on a large scale and employs commercially/readily available starting reagents.

Herein we report the isolation and characterization of a novel series of mixed-valent, heteroleptic transition metal diketonates that can be utilized as single-source precursors for the low-temperature preparation of oxide materials. Homometallic parent compounds [Fe^III^(acac)_3_][Fe^II^(hfac)_2_] (**1**) and [Fe^II^(hfac)_2_][Fe^III^(acac)_3_][Fe^II^(hfac)_2_] (**2**) (acac = acetylacetonate; hfac = hexafluoroacetylacetonate) with dinuclear and trinuclear molecular structures, respectively, obtained in the course of this work, have been utilized as templates for the design of new mixed-valent heterometallic single-source precursors [Fe^III^(acac)_3_][Mn^II^(hfac)_2_] (**4**) and [Ni^II^(hfac)_2_][Fe^III^(acac)_3_][Ni^II^(hfac)_2_] (**5**). The combination of two different diketonate ligands with electron-donating (acac) and electron-withdrawing (hfac) substituents was found to be crucial for maintaining the above mixed-valent heterometallic assemblies.

## Results and discussion

As we have previously stated,^20^ heterometallic diketonates can be obtained by the reaction of corresponding homometallic constituents when at least one of those is coordinatively unsaturated or is capable of producing coordinatively unsaturated fragments upon dissociation in the gas phase or in solution. A clear illustration of this statement is a family of heterometallic bismuth–transition metal diketonates, Bi_2_M(β-dik)_8_ (M = Mn–Zn),^21^ in which only coordinatively unsaturated M^II^(β-dik)_2_ units participate in bridging interactions with Bi-chelating diketonate oxygens, thus holding the trinuclear molecule together, while Bi^III^(β-dik)_3_ fragments do not require any additional contacts. In accord with this example, the mixed-valent transition metal diketonates that consist of coordinatively saturated M^III^(β-dik)_3_ octahedral units and coordinatively unsaturated M^II^(β-dik)_2_ fragments should be capable of forming the polynuclear molecular assemblies. However, until now, we were unable to obtain such mixed-valent transition metal diketonates. In this work, we applied a mixed-ligand approach to synthesize the target molecules. It was reasoned that transition metal(ii) center responsible for forming bridging interactions with oxygen atoms of neighboring unit/units should be highly Lewis acidic and therefore be chelated by diketonates with electron-withdrawing groups, such as fluorinated hfac (hexafluoroacetylacetonate). On the other hand, the transition metal(iii) counterpart should have sterically uncongested ligands with electron-donating substituents such as acac (acetylacetonate) in order to make its diketonate oxygen atoms more attractive for participating in bridging interactions with M^II^ center.

Iron was thought as the best candidate among other transition metals to explore the isolation of mixed-valent diketonates. Iron(ii) hexafluoroacetylacetonate seems the most suited to produce coordinatively unsaturated [Fe(hfac)_2_] fragments in the gas phase as well as to coordinate to electron-rich diketonate oxygens from the [Fe(acac)_3_] unit. Homometallic diketonate Fe(hfac)_2_ has a unique dinuclear structure that is held together by four very long Fe···O interactions of 2.87–3.02 Å, while other first-row transition metal compounds M(hfac)_2_ consist of trinuclear molecules that feature strong bridging M–O bonds of 2.20 (6×, Mn), 2.09–2.42 (6×, Co), and 2.06–2.43 Å (6×, Ni).^22^

Mixed-valent heteroleptic iron diketonates with two different Fe^iii^/Fe^ii^ ratios have been obtained by the solid state/gas phase stoichiometric reactions between Fe(acac)_3_ and Fe(hfac)_2_ in sealed evacuated ampules (for synthetic details, see ESI, Table S1†):

1 Fe(acac)_3_ + Fe(hfac)_2_ → Fe_2_(acac)_3_(hfac)_2_ (**1**)

2 Fe(acac)_3_ + 2Fe(hfac)_2_ → Fe_3_(acac)_3_(hfac)_4_ (**2**)

Iron diketonate complexes **1** and **2** were collected as red crystalline materials from the cold end of the containers with nearly quantitative yields. The purity of the products was confirmed by comparison of the X-ray powder diffraction patterns with theoretical ones calculated on the basis of single crystal data (ESI, Fig. S1 and S2†). Compounds are highly volatile (above 75 °C) and can be quantitatively resublimed at 95 °C. The traces of thermal decomposition in sealed ampules become noticeable above 100 °C. Products are readily soluble in noncoordinating (CH_2_Cl_2_, CHCl_3_, hexanes, benzene) as well as in coordinating (acetone, THF, and DMSO) solvents. Complexes **1** and **2** are relatively stable in moist air and can be handled outside the glove box for a reasonable period of time in the course of characterization and decomposition studies. Such behavior strikingly contrasts the sensitivity of one of their components, Fe(hfac)_2_, that immediately hydrolyzes/oxidizes in open air and should only be used as freshly prepared.

**Fig. 1:**
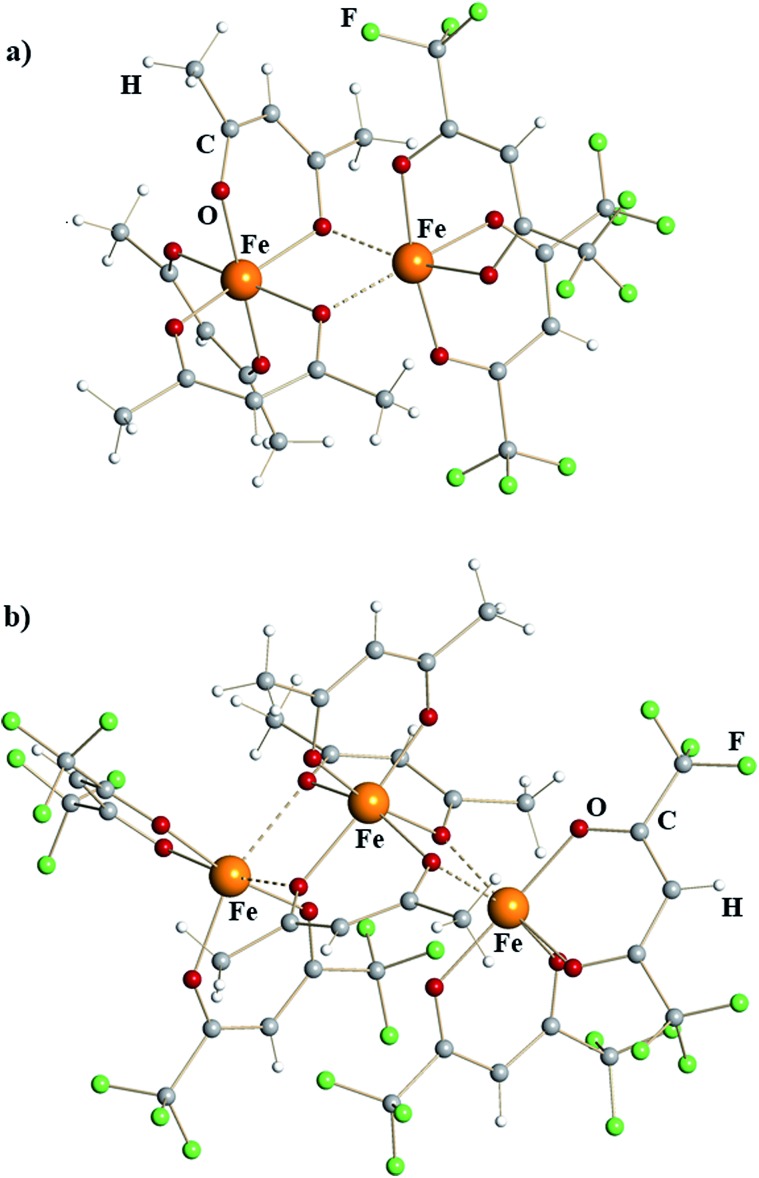
Molecular structures of mixed-valent iron β-diketonates (a) [Fe^III^(acac)_3_][Fe^II^(hfac)_2_] (**1**) and (b) [Fe^II^(hfac)_2_][Fe^III^(acac)_3_][Fe^II^(hfac)_2_] (**2**). Atoms are represented by spheres of arbitrary radii. Bridging Fe–O interactions are shown as dashed lines.

X-ray structural investigation of mixed-valent iron diketonates revealed dinuclear molecule [Fe(acac)_3_][Fe(hfac)_2_] (**1**) and trinuclear molecule [Fe(hfac)_2_][Fe(acac)_3_][Fe(hfac)_2_] (**2**) (Fig. 1). Complex **1** is a typical edge-sharing bioctahedral molecule with three chelating acac ligands on one iron atom and two chelating hfac groups on the other. The latter completes its octahedral environment by making two additional interactions with oxygen atoms of acac. Analysis of the Fe–O bonds (ESI, Table S4†) shows that the coordinatively saturated, *tris*-chelated iron center in **1** is essentially the same as in the structure of Fe(acac)_3_^23^ (2.00 *vs.* 1.99 Å, Table 1). There are, as expected, subtle differences between Fe–O bonds for purely chelating (1.98 Å) and chelating-bridging oxygens (2.04 Å). The second iron atom in dinuclear complex **1** has two chelating hfac ligands (Fe–O_av_ = 2.07 Å) and two additional *cis*-bridging interactions with oxygen atoms of acac (2.18 Å). This coordination environment can be compared with that of Fe^II^ atom in recently reported^14*d*^ heterometallic tetranuclear complex Pb_2_Fe_2_(acac)_2_(hfac)_6_, in which [Fe(hfac)_2_] fragments (Fe–O_av_ = 2.05 Å) have two additional *cis*-interactions with Pb-chelating acac oxygens (2.19 Å). In trinuclear structure of **2**, the central [Fe(acac)_3_] fragment is sandwiched between two [Fe(hfac)_2_] units, thus offering four acac oxygens for bridging interactions. Two acac groups act as chelating-bridging through both oxygens, while the third ligand remains purely chelating. The central *tris*-chelated unit is similar to that in **1** with Fe–O bonds averaged at 1.96 and 2.03 Å for chelating and chelating-bridging ligands, respectively (Table 1). The metal–ligand distances in two *bis*-chelated [Fe(hfac)_2_] fragments are also comparable to the corresponding characteristics in **1**, measured at 2.05/2.04 Å for chelating oxygens and 2.19/2.25 Å for *cis*-bridging interactions with the central unit.

**Table 1:** Averaged M–O distances (Å) in polynuclear diketonates **1–5** and in related compounds

	M^III^–O_c_^*a*^	M^III^–O_c–b_^*b*^	M^II^–O_c_^*a*^	M^II^–O_b_^*c*^
[Fe^III^(acac)_3_][Fe^II^(hfac)_2_] (**1**)	1.981(2)	2.035(2)	2.073(2)	2.183(2)
Fe^III^(acac)_3_ (ref. 23)	1.991(3)			
[Pb(acac)(hfac)]_2_[Fe^II^(hfac)_2_]_2_ (ref. 14*d*)			2.051(3)	2.192(3)
[Fe^II^(hfac)_2_][Fe^III^(acac)_3_][Fe^II^(hfac)_2_] (**2**)	1.958(2)	2.026(2)	2.051(2)/2.043(2)^*d*^	2.192(2)/2.245(2)^*d*^
[Fe^II^(hfac)_2_][Fe^III^(acac)_2_(hfac)][Fe^II^(hfac)_2_] (**3**)	2.008(2)^*e*^	1.999(2)	2.042(2)	2.233(2)
[Fe^II^(hfac)_2_]_2_ (ref. 22)				2.871(4), 3.018(4)
[Fe^III^(acac)_3_][Mn^II^(hfac)_2_] (**4**)	1.977(2)	2.037(2)	2.126(2)	2.204(2)
Mn^III^(acac)_3_ (ref. 24)	1.9349(2), 2.1108(3)^*f*^			
[Pb(hfac)_2_][Mn^II^(hfac)_2_]_∞_ (ref. 14*a*)			2.118(4)	2.231(4)
[Ni^II^(hfac)_2_][Fe^III^(acac)_3_][Ni^II^(hfac)_2_] (**5**)	1.959(2)	2.026(2)	2.009(2)/2.010(2)^*d*^	2.133(2)/2.179(2)^*d*^
[NaNi^II^(hfac)_3_]_∞_ (ref. 25)			2.026(5)^*g*^	
[NaFe^II^(hfac)_3_]_∞_ (ref. 25)			2.075(2)^*g*^	

^*a*^Chelating.

^*b*^Chelating-bridging.

^*c*^Bridging.

^*d*^For two [M(hfac)_2_] fragments.

^*e*^M–O_hfac_.

^*f*^1.9349 (4×), 2.1108 (2×) Å.

^*g*^All diketonates are chelating-bridging.

Based on nearly quantitative reaction yields and analysis of iron–oxygen bonds, the title complexes can be formulated as [Fe^III^(acac)_3_][Fe^II^(hfac)_2_] (**1**) and [Fe^II^(hfac)_2_][Fe^III^(acac)_3_][Fe^II^(hfac)_2_] (**2**). To the best of our knowledge, they represent the first structurally characterized examples of mixed-valent *homometallic* transition metal diketonates. In both complexes, the Lewis acidic, coordinatively unsaturated Fe^II^ centers, chelated by two electron-withdrawing hfac groups, maintain bridging interactions with oxygen atoms of electron-donating acac ligands attached to the neighboring [Fe^III^(acac)_3_] unit. In accord with our initial expectations, iron indeed appeared as the best candidate among the first-row transition metals to isolate mixed-valent diketonates. Products **1** and **2** are capable of providing considerably stronger Fe–O interactions (2.18–2.25 Å) for coordinatively unsaturated iron center in [Fe(hfac)_2_] fragments compared to those in the dimeric structure of parent [Fe(hfac)_2_]_2_ reagent (2.87–3.02 Å).^22^

When we tried to switch the ligands on starting reagents and to run stoichiometric reaction between Fe(hfac)_3_ and Fe(acac)_2_, it resulted in yet another polynuclear homometallic diketonate [Fe(hfac)_2_][Fe(acac)_2_(hfac)][Fe(hfac)_2_] (**3**). Complex **3** was identified as a major product of the reaction that proceeds according to the equation:

3 2Fe(acac)_2_ + 2Fe(hfac)_3_ → Fe_3_(acac)_2_(hfac)_5_ + Fe(acac)_2_(hfac)

The formation of complex **3** can be explained by a prompt ligand exchange between Fe^II^ and Fe^III^ centers, the event that is rather common in metal diketonate chemistry. Alternative pathway for the formation of compound **3** is an instantaneous electron transfer upon formation of the diketonate bridge. Such intramolecular redox process is well established for the Fe^II^/Fe^III^ cyanide complexes.^26^

Molecular structure of diketonate **3** (ESI, Fig. S7 and Table S6†) is very similar to that of **2** with a sole exception of one hfac ligand chelating the central Fe^III^ atom. Remarkably, this ligand with electron-withdrawing groups does not participate in bridging interactions with Fe^II^ centers on both sides of the molecule and remains purely chelating. Subsequent theoretical modeling of the possible adducts of M^II^(acac)_2_ with M^III^(hfac)_3_ at the DFT level (PBE0/def2-TZVP^27^) revealed that compounds of the formula [M^III^(hfac)_3_][M^II^(acac)_2_] do not correspond to the local minima on the potential energy surfaces. Optimization procedure eventually converged to the systems in which the initial homometallic fragments are held together by weak non-covalent interactions (ESI, Fig. S13 and S14, Tables S11 and S12†).

In the course of this work, mixed-valent heteroleptic iron diketonates were envisioned as synthetic platforms for the preparation of heterobimetallic species. Complexes **1** and **2** have been used as templates to design target heterometallic (transition metal-transition metal) diketonates with M : M′ = 1 : 1 and 1 : 2 ratios that can be employed as single-source precursors for the synthesis of corresponding mixed-metal oxide materials. Mixed-valent heteroleptic *heterometallic* diketonates have been obtained by the solid state/gas phase stoichiometric reactions similar to those in eqn (1) and (2) by using Fe(acac)_3_ and M(hfac)_2_ (M = Mn, Ni) as starting reagents (ESI, Table S1†):

4 Fe(acac)_3_ + Mn(hfac)_2_ → FeMn(acac)_3_(hfac)_2_ (**4**)

5 Fe(acac)_3_ + 2Ni(hfac)_2_ → FeNi_2_(acac)_3_(hfac)_4_ (**5**)

The products **4** and **5** were collected as red-brown block-shaped crystals from the cold end of the ampules with nearly quantitative yields. Elemental analysis confirmed the metal ratios as Fe : Mn = 1.03 : 0.97 for **4** and Fe : Ni = 0.96 : 2.04 for **5**. The purity of the products was further verified by comparison of the experimental X-ray powder diffraction patterns with theoretical profiles calculated on the basis of single crystal data (ESI, Fig. S3 and S4†). Both compounds are highly volatile and can be quantitatively resublimed at 95 (**4**) and 105 °C (**5**). Decomposition of crystals in a sealed ampule is visible above 100 (**4**) and 110 °C (**5**). Heterometallic complexes are readily soluble in a variety of common solvents and are moderately stable in moist air.

**Fig. 2:**
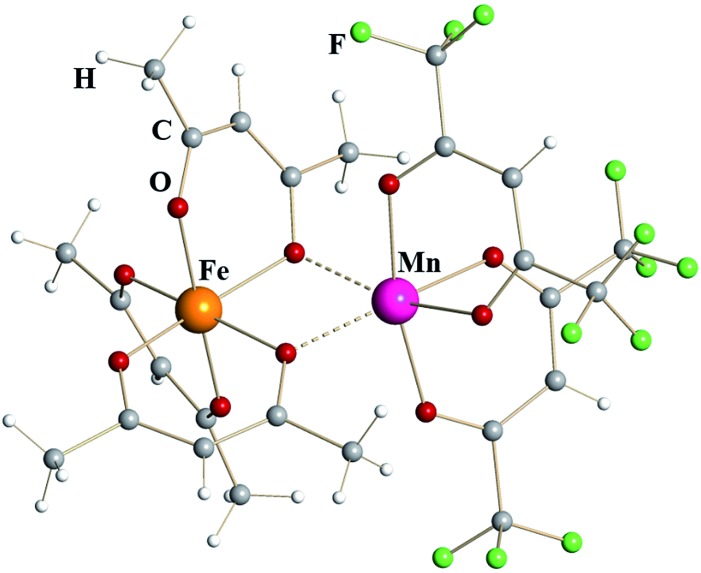
Dinuclear structure of heterometallic β-diketonate [Fe^III^(acac)_3_][Mn^II^(hfac)_2_] (**4**). Atoms are represented by spheres of arbitrary radii. Bridging Mn–O interactions are shown as dashed lines.

Iron–manganese heterometallic diketonate [Fe(acac)_3_][Mn(hfac)_2_] (**4**) (Fig. 2) appears isomorphous to [Fe(acac)_3_][Fe(hfac)_2_] (**1**), though the unit cell parameters (ESI, Table S3†) are noticeably different. The *tris*-acac chelated metal fragment is identical to that of Fe^III^ in complex **1** with M–O distances to chelating and chelating-bridging oxygens being 1.98 and 2.04 Å, respectively (Table 1). This metal atom was therefore identified as trivalent Fe. Were this site occupied by Mn, one should expect a Jahn–Teller distortion similar to that in Mn^III^(acac)_3_, which features four short equatorial (1.93 Å) and two long axial (2.11 Å) metal–oxygen bonds.^24^ The *bis*-hfac chelated metal center is clearly different from the one in homometallic complex **1**. It displays slightly longer bridging interactions (2.20 Å) and significantly longer chelating M–O bonds (2.13 Å). Such coordination environment is similar to that in [Mn^II^(hfac)_2_] fragment of heterometallic diketonate PbMn(hfac)_4_^14*a*^ that also has two additional *cis*-interactions with oxygen atoms of neighboring diketonates (2.23 and 2.12 Å for bridging and chelating oxygens, respectively). Based on these geometrical considerations as well as on the results of elemental analysis, this atom was identified as divalent Mn, thus giving the formulation of complex **4** as [Fe^III^(acac)_3_][Mn^II^(hfac)_2_]. Refinement of the crystal structure of **4** as [Mn^III^(acac)_3_][Fe^II^(hfac)_2_] (**4′**) does not lead to a meaningful increase in the *R*-value (0.0468 *vs.* 0.0444), however it results in profound disparity of thermal parameters for the M^III^ and M^II^ sites (0.0130/0.0202 *vs.* 0.0164/0.0164 Å^2^ for **4**). It is worth noting that the reaction of Mn(acac)_3_ with Fe(hfac)_2_ does not produce, at least in our hands, either compound **4** by redox process coupled with ligands exchange or stoichiometric complex **4′**. The reaction is apparently taking place, since no volatile products (including starting reagents) are detected in the cold zone, however at elevated temperatures the appearance of melt and oily residues was observed resulting in no crystalline materials upon cooling the vessel down.

In order to provide additional support for the metal site assignment, a theoretical investigation of two possible “isomers”, namely [Fe^III^(acac)_3_][Mn^II^(hfac)_2_] (**4**) and [Mn^III^(acac)_3_][Fe^II^(hfac)_2_] (**4′**), was performed using PBE0/def2-TZVP approach (ESI, Fig. S11 and S12, Tables S9 and S10†). Both molecules were found to be local minima on the corresponding potential energy surfaces. The system **4** was calculated to be *ca.* 8.54 kcal mol^–1^ more stable than system **4′** at this level of theory. However, it is known that, albeit offering a good agreement between experimental and calculated geometrical parameters, the standard hybrid functionals sometimes lack accuracy in providing an evaluation of energetics. In order to obtain more reliable energetics, a newly developed DFT approach called double-hybrid technique with empirical dispersion corrections (here B2PLYP-D3^28^) was employed.^29^ Importantly, this approach was also combined with relativistically re-contracted full-electron basis sets of triple-*ζ* quality (TZVP). The relativistic effects were accounted explicitly through the ZORA approximation.^30^ Applied to the target systems, this technique also gave a preference of 9.78 kcal mol^–1^ for the molecule **4**. The latter implies the formation of adduct **4′** as thermodynamically unfavorable, which is in a full agreement with our experimental observations.

**Fig. 3:**
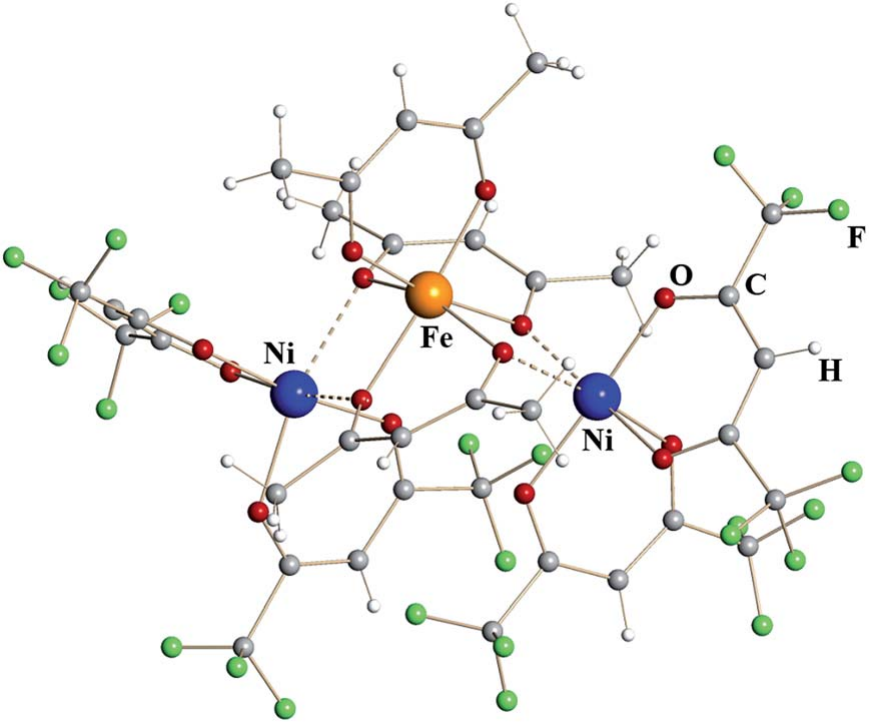
Trinuclear structure of heterometallic β-diketonate [Ni^II^(hfac)_2_][Fe^III^(acac)_3_][Ni^II^(hfac)_2_] (**5**). Atoms are represented by spheres of arbitrary radii. Bridging Ni–O interactions are shown as dashed lines.

Trinuclear iron–nickel heterometallic diketonate **5** (Fig. 3) is isomorphous to the corresponding iron diketonate **2**. The central *tris*-acac coordinated metal fragment with M–O distances to chelating (1.96 Å) and to chelating-bridging (2.03 Å) oxygens is identical to that in complex **2** (Table 1). Based on these considerations and the fact that Ni^III^ diketonates are unknown, this metal site was identified as trivalent Fe. The *bis*-hfac chelated metal fragments flanking the central unit are noticeably different from those in complex **2**. They exhibit both shorter bridging interactions (2.13/2.18 Å) and shorter chelating M–O distances (2.01 Å). The latter is typical when comparing isomorphous hfac complexes of divalent nickel with iron.^25^ Taking into account the results of elemental analysis, these atoms were assigned as divalent Ni. Thus, heterometallic complex **5** can be formulated as [Ni^II^(hfac)_2_][Fe^III^(acac)_3_][Ni^II^(hfac)_2_].

Isomorphous pairs **1**/**4** and **2**/**5** consist of two or three edge-sharing octahedral metal units, respectively, with every metal center chelated by two or three diketonate ligands. Therefore, such units are all chiral and exist as *Δ*- and *Λ*-enantiomers. Dinuclear complexes [Fe(acac)_3_][Fe(hfac)_2_] (**1**) and [Fe(acac)_3_][Mn(hfac)_2_] (**4**) crystallize in centrosymmetric space group *P*ī and feature a pair of *Δ*,*Δ*- and *Λ*,*Λ*-enantiomers in the unit cell (ESI, Fig. S10†). Trinuclear complexes [Fe(hfac)_2_][Fe(acac)_3_][Fe(hfac)_2_] (**2**) and [Ni(hfac)_2_][Fe(acac)_3_][Ni(hfac)_2_] (**5**) crystallize in the *Sohncke* space group *P*2_1_ and were found to contain only *Λ*,*Λ*,*Λ*-enantiomers. After checking a number of crystals we were able to find a pure *Δ*,*Δ*,*Δ*-enantiomer (**2a**) that, as expected, conforms to the same unit cell parameters as complex **2** (ESI, Table S3a†). Interestingly, the related trinuclear compound [Fe(hfac)_2_][Fe(acac)_2_(hfac)][Fe(hfac)_2_] (**3**) crystallizes in centrosymmetric *C*2/*c* space group (ESI, Table S3a†) and contains two pairs of *Δ*,*Δ*,*Δ*- and *Λ*,*Λ*,*Λ*-enantiomers in the unit cell. It is worth noting, that no diastereomers were observed for both dinuclear and trinuclear molecules after checking a total of more than 30 crystals of different compounds. This interesting finding is currently a subject of a separate investigation.

**Fig. 4:**
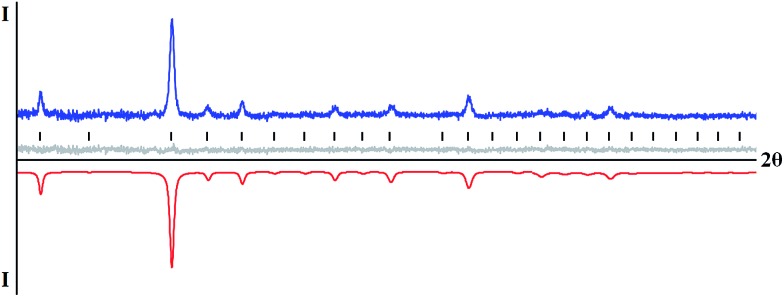
X-ray powder diffraction pattern of M_2_O_3_ type oxide obtained by thermal decomposition of [Fe(acac)_3_][Mn(hfac)_2_] (**4**) precursor at 500 °C and the Le Bail fit for the cubic unit cell (Sp. gr. *Ia*3, *a* = 9.408(2) Å). The blue and red curves are experimental and calculated patterns, respectively. The gray line is a difference curve. Theoretical peak positions are shown in the middle as black lines.

**Fig. 5:**
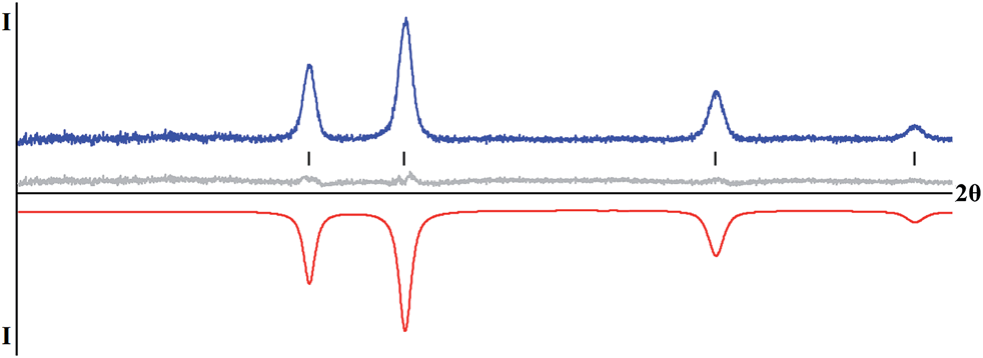
X-ray powder diffraction pattern of MO type oxide obtained by thermal decomposition of [Ni(hfac)_2_][Fe(acac)_3_][Ni(hfac)_2_] (**5**) precursor at 500 °C and the Le Bail fit for the cubic unit cell (sp. gr. *Fm*3*m*, *a* = 4.179(1) Å). The blue and red curves are experimental and calculated patterns, respectively. The gray line is a difference curve. Theoretical peak positions are shown in the middle as black lines.

Thermal decomposition of mixed-valent diketonate complexes has been investigated. According to our observations, complexes **1–5** start to decompose at relatively low temperatures, just over 100 °C. Homometallic complex [Fe(acac)_3_][Fe(hfac)_2_] (**1**) was confirmed by X-ray powder diffraction to yield Fe_2_O_3_ oxide upon thermal decomposition in air. The phase-pure rhombohedral hematite^31^ can be clearly identified in X-ray powder pattern of decomposition residues obtained at 500 °C (ESI, Fig. S19†). Thermal decomposition of heterometallic precursors [Fe(acac)_3_][Mn(hfac)_2_] (**4**) and [Ni(hfac)_2_][Fe(acac)_3_][Ni(hfac)_2_] (**5**) in air at low temperatures (below 300 °C) results in amorphous phases (ESI, Fig. S20 and S21†). Increasing decomposition temperature to 500 °C leads to crystalline residues that were identified by X-ray powder diffraction as M_2_O_3_ type oxide for the Fe–Mn diketonate precursor **4** (Fig. 4) and as MO type oxide for the Fe–Ni complex **5** (Fig. 5).

The Fe_2_O_3_ (maghemite)^32^ and Mn_2_O_3_ (bixbyite)^33^ phases are isomorphous with very close unit cell parameters of the body-centered unit cells. Those oxides, as well as mixed-metal phases like FeMnO_3_,^34^ cannot be distinguished by their X-ray powder diffraction patterns. The decomposition residues were analyzed by taking high angle annular dark field scanning transmission electron microscopy (HAADF-STEM) images and energy dispersive X-ray (EDX) spectra (ESI, Fig. S22 and S23†). The analysis revealed that the sample consists of two phases. One of those is an Fe^III^ oxide that appears in the form of micron-sized crystals. Another one is the mixed Fe–Mn oxide that forms highly agglomerated nanocrystals. The Fe : Mn atomic ratio in the latter solid solution species varies significantly around the average 26(9) : 74(9) value. Further increase of the annealing temperature and elongation of annealing time resulted in the well-known^35^ transformation of cubic Fe_2_O_3_ to rhombohedral hematite modification, while mixed-metal oxide with increased crystallinity remains in a mixture as a major phase (ESI, Fig. S20†). In the case of Fe–Ni precursor **5**, the increase of decomposition temperature to 500 °C is accompanied by an appearance of a crystalline phase that is closer to both NiO^36^ and nickel-rich Fe–Ni oxides,^37^ rather than to FeO (ESI, Fig. S21†). TEM analysis confirmed that the residue consists of highly agglomerated crystalline Fe–Ni oxide nanoparticles having the NiO-type face-centered cubic structure with *a* ≈ 4.3 Å. The average Fe : Ni atomic ratio is 19(3) : 81(3), while iron is not homogeneously distributed and demonstrates a tendency towards segregation at the surface of the nanoparticles (ESI, Fig. S24–S29†). Increasing the decomposition temperature to 700 °C, results in improved crystallinity of the MO phase as well as in partial oxidation of iron and appearance of Fe_3_O_4_ (or Fe_*x*_Ni_3–*x*_O_4_) impurities (ESI, Fig. S21†).

## Conclusions

The first mixed-valent iron β-diketonates with different Fe^III^/Fe^II^ ratios have been synthesized by applying the mixed-ligand approach. In these heteroleptic complexes, the Lewis acidic, coordinatively unsaturated Fe^II^ centers chelated by two ligands with electron-withdrawing substituents maintain bridging interactions with oxygen atoms of electron-donating diketonates that chelate the neighboring Fe^III^ atoms. Homometallic parent molecules have been used as templates to obtain heterometallic mixed-valent Fe^III^ : Mn^II^ = 1 : 1 and Fe^III^ : Ni^II^ = 1 : 2 diketonates. We have shown that the first-row transition metal sites in the above heterobimetallic molecules can be unambiguously identified based solely on single crystal X-ray diffraction data. The mixed-valent/mixed-ligand approach designed in this work opens broad opportunities for the synthesis of heterometallic (transition metal – transition metal) diketonates with different M : M′ ratios. A number of other metal combinations may be explored, specifically those including second- and third-row transition metals that are known to prefer trivalent diketonate complexes with electron-donating acac ligands.^38^ Heterometallic complexes obtained in the course of this study have been found to act as effective single-source precursors for the synthesis of mixed-transition metal oxide materials M_*x*_M′2–*x*O_3_ and M_*x*_M′1–*x*O. Highly volatile precursors having low decomposition temperatures can be effectively utilized for the preparation of both amorphous and crystalline heterometallic oxides in the form of thin films or nanosized particles that are known to operate as efficient catalysts in oxygen evolution reaction.

## Supplementary Material

Supplementary information

Crystal structure data
