# Supplementary material for: Mixed-valent, heteroleptic homometallic diketonates as templates for the design of volatile heterometallic precursors
Source: Chem Sci. 2015 Feb 25;6(5):2835–42. doi: 10.1039/c4sc04002c (PMC5654368; doi:10.1039/c4sc04002c)
Supplement: Supplementary file 1 [file SC-006-C4SC04002C-s001.pdf]

Electronic Supplementary Material (ESI) for Chemical Science.
This journal is © The Royal Society of Chemistry 2015

Electronic Supplementary Information

Mixed-Valent, Heteroleptic Homometallic Diketonates as Templates for the Design of Heterometallic Precursors

Craig M. Lieberman,[a] Alexander S. Filatov,[a] Zheng Wei,[a] Andrey Yu. Rogachev,[b] Artem M. Abakumov,[c] and Evgeny V. Dikarev[a]*

[a]*Department of Chemistry, University at Albany, Albany, NY 12222, USA*

[b]*Department of Biological and Chemical Sciences, Illinois Institute of Technology, Chicago, IL 60616, USA*

[c]*EMAT, University of Antwerp, Groenenborgerlaan 171, B-2020 Antwerp, Belgium*

*Author to whom correspondence should be addressed. E-mail: edikarev@albany.edu. Phone: (518)442-4401. Fax: (518)442-3462.

Content

Experimental Section. General Procedures……………………………………………………ESI3
Synthesis of Homometallic and Heterometallic Diketonate Precursors **1-5**……………….....ESI4
X-ray Powder Diffraction Patterns of Homo- and Heterometallic Diketonates…..……..........ESI5
Crystal Growth……………………………….………………………….....………......ESI8
X-ray Crystallographic Procedures…………………………………………….……...……ESI8
Molecular Structures of Homo- and Heterometallic Diketonates **1-5**…………………..…...…ESI11
Theoretical Calculations……………………………………………………………..ESI24
ATR-IR Spectra of Diketonate Precursors……..……..…………………...………………ESI34
Analysis of Decomposition Residues of Diketonate Precursors………..…...……………….ESI36
References………………………………………………………………………………ESI46

Experimental Section. General Procedures

All of the manipulations were carried out in a dry, oxygen-free argon atmosphere by employing standard Schlenk and glove box techniques. Transition metal(III) diketonates, $M(acac)_3$ (M = Fe, Mn), hexafluoroacetylacetone (Hhfac), iron and manganese metals were purchased from Sigma-Aldrich and used upon received. Transition metal(III) and metal(II) diketonates $M(hfac)_3$ (M = Fe, Mn) and $M(hfac)_2$ (M = Fe, Mn, Ni), respectively, were prepared according to previously published procedures.[1,2] The attenuated total reflection (ATR) spectra were recorded on a PerkinElmer Spectrum 100 FT-IR spectrometer. Thermal decomposition of heterometallic precursors was studied in air at ambient pressure. The solid sample (*ca.* 40 mg) was placed into a 20 mL Coors high-alumina crucible (Aldrich) and heated at a rate of *ca.* 35 °C/min in a muffle furnace (Lindberg Blue M). The decomposition residues were analyzed by X-ray powder diffraction. X-ray powder diffraction data were collected on a Bruker D8 Advance diffractometer (Cu Kα radiation, focusing Göbel Mirror, LynxEye one-dimensional detector, step of 0.02^o 2θ, 20 °C). The crystalline samples under investigation were ground and placed in the dome-like airtight zero-background holders inside a glove box. Le Bail fit for powder diffraction patterns has been performed using TOPAS, version 4 software package (Bruker AXS, 2006). Elemental analysis has been done by Complete Analysis Laboratories, Inc., Parsippany, NJ. Samples for transmission electron microscopy (TEM) measurements were prepared by crushing the decomposition residues in a mortar in ethanol and depositing drops of suspension onto holey carbon grids. Bright field TEM images, high angle annular dark field scanning transmission electron microscopy (HAADF-STEM) images and energy dispersive X-ray (EDX) spectra were obtained with a Tecnai Osiris electron microscope operated at 200 kV.

Synthesis of Homometallic and Heterometallic Diketonate Precursors 1-5

Homo- and heterometallic diketonates **1-5** have been obtained by the solid state synthesis. In a typical experiment, a mixture of two powdered reagents was sealed in an evacuated glass ampule and placed in an electric furnace having a temperature gradient along the length of the tube. The ampule was kept at constant temperature for several days to allow crystals of diketonate products **1-5** to be deposited in the cold section of the container where the temperature was set approximately 5 °C lower. The yield was calculated in each case based on amount of crystals collected. Anal. Calc. for $FeMnC_{25}H_{23}O_{10}F_{12}$ (**4**) (%): Fe 6.79; Mn 6.78; C 36.52; H 2.82. Found: Fe 7.01; Mn 6.41; C 36.30; H 2.52. Anal. Calc. for $FeNi_2C_{35}H_{25}O_{14}F_{24}$ (**5**) (%): Fe 4.30; Ni 9.04; C 32.37; H 1.94. Found: Fe 3.98; Ni 8.92; C 32.21; H 1.62. The experimental conditions for different synthetic methods are summarized in the Table S1.

Table S1. Experimental Conditions for the Solid State Syntheses of [Fe(acac)$_3$][Fe(hfac)$_2$] (**1**), [Fe(hfac)$_2$][Fe(acac)$_3$][Fe(hfac)$_2$] (**2**), [Fe(hfac)$_2$][Fe(acac)$_2$(hfac)][Fe(hfac)$_2$] (**3**), [Fe(acac)$_3$][Mn(hfac)$_2$] (**4**), and [Ni(hfac)$_2$][Fe(acac)$_3$][Ni(hfac)$_2$] (**5**)

Reagent I g/mmol	Reagent II g/mmol	Temp. (^{o}C)	Time (days)	Yield (%)
**[Fe(acac)$_3$][Fe(hfac)$_2$]** (**1**) (red block-shaped crystals)				
Fe(acac)$_3$ 0.064/0.18	Fe(hfac)$_2$ 0.084/0.18	85-95	4-6	95
**[Fe(hfac)$_2$][Fe(acac)$_3$][Fe(hfac)$_2$]** (**2**) (red block-shaped crystals)				
Fe(acac)$_3$ 0.032/0.09	Fe(hfac)$_2$ 0.084/0.18	85-95	4-5	85
**[Fe(hfac)$_2$][Fe(acac)$_2$(hfac)][Fe(hfac)$_2$]** (**3**) (red block-shaped crystals)				
Fe(hfac)$_3$ 0.016/0.023	Fe(acac)$_2$ 0.06/0.023	85	4	50
**[Fe(acac)$_3$][Mn(hfac)$_2$]** (**4**) (red-brown block-shaped crystals).				
Fe(acac)$_3$ 0.064/0.18	Mn(hfac)$_2$ 0.084/0.18	85-95	5-7	90
**[Ni(hfac)$_2$][Fe(acac)$_3$][Ni(hfac)$_2$]** (**5**) (red-brown block-shaped crystals)				
Fe(acac)$_3$ 0.032/0.09	Ni(hfac)$_2$ 0.086/0.18	90-105	5-7	85

X-ray Powder Diffraction Patterns of Homo- and Heterometallic Diketonates

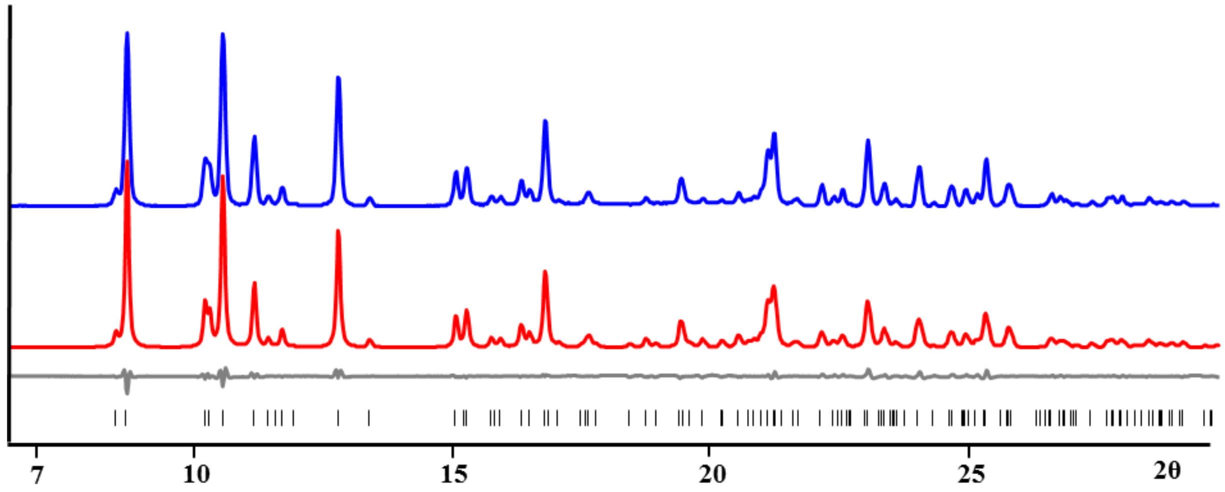

Figure S1. X-ray powder pattern of [Fe(acac)$_3$][Fe(hfac)$_2$] (**1**) and the Le Bail fit. The blue and red curves are the experimental and calculated patterns, respectively. The grey line is the difference curve. Theoretical peak positions are shown at the bottom as black lines.

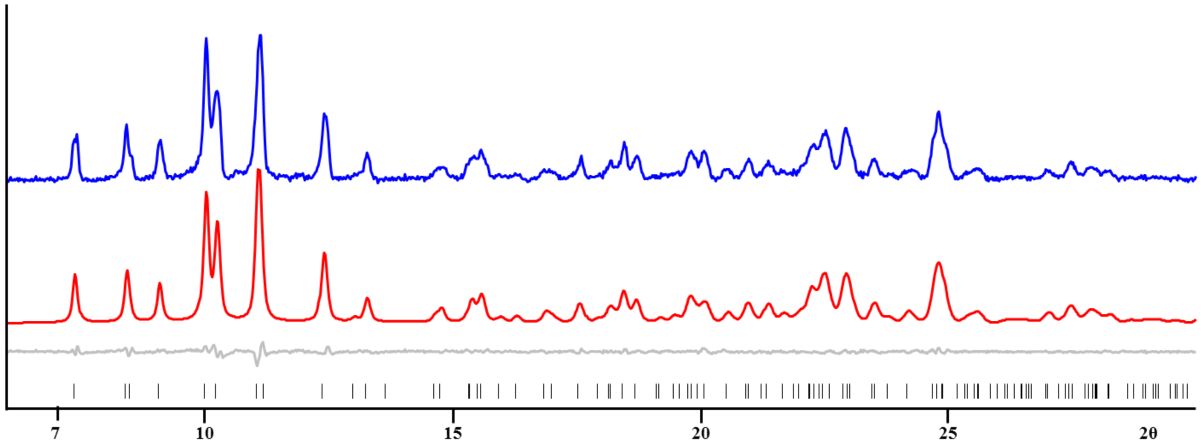

Figure S2. X-ray powder pattern of [Fe(hfac)$_2$][Fe(acac)$_3$][Fe(hfac)$_2$] (**2**) and the Le Bail fit. The blue and red curves are the experimental and calculated patterns, respectively. The grey line is the difference curve. Theoretical peak positions are shown at the bottom as black lines.

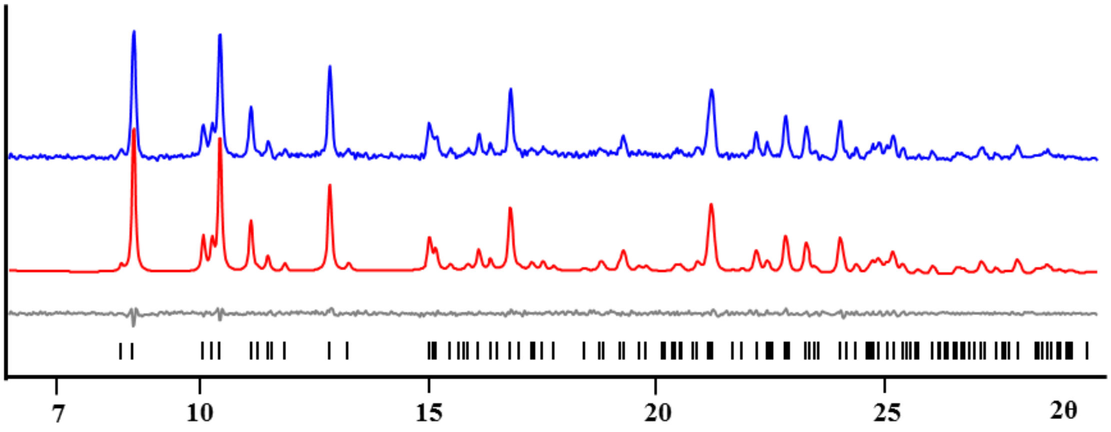

Figure S3. X-ray powder pattern of $[Fe(acac)_3][Mn(hfac)_2]$ (**4**) and the Le Bail fit. The blue and red curves are the experimental and calculated patterns, respectively. The grey line is the difference curve. Theoretical peak positions are shown at the bottom as black lines.

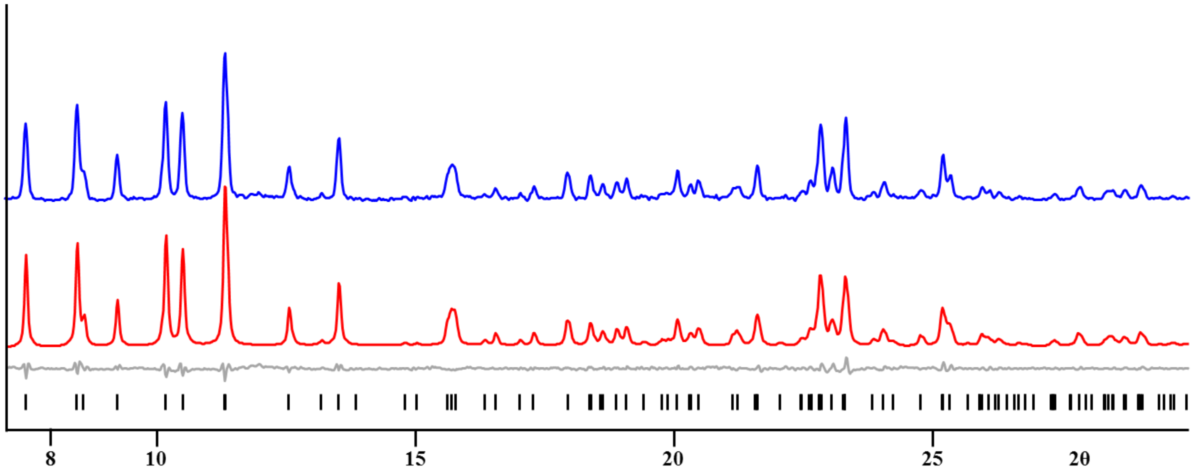

Figure S4. X-ray powder pattern of $[Ni(hfac)_2][Fe(acac)_3][Ni(hfac)_2]$ (**5**) and the Le Bail fit. The blue and red curves are the experimental and calculated patterns, respectively. The grey line is the difference curve. Theoretical peak positions are shown at the bottom as black lines.

Table S2. Unit Cell Parameters for Homo- and Heterometallic Diketonates **1**, **2**, **4**, and **5** Obtained by the Le Bail Fit and from the Single Crystal Data

Compound	Sp. Gr.	Unit Cell Parameters (Å, deg., $Å^3$)	
		Le Bail fit (20 °C)	Single crystal (-173 °C)
$[Fe(acac)_3][Fe(hfac)_2]$ (**1**)	*P*-1	*a* = 9.5084(7)	*a* = 9.507(2)
		b = 11.7343(7)	*b* = 11.729(3)
		c = 16.6103(7)	*c* = 16.605(4)
		α = 102.688(6)	*α* = 102.703(2)
		β = 96.779(1)	*β* = 96.771(2)
		γ = 112.585(7)	*γ* = 112.595(2)
		V = 1626.7(1)	*V* = 1625.0(7)
$[Fe(hfac)_2][Fe(acac)_3][Fe(hfac)_2]$ (**2**)	$P2_1$	*a* = 10.0323 (4)	*a* = 10.0494(10)[a]
		b = 21.0176 (7)	*b* = 21.0159 (20)
		c = 12.3594(5)	*c* = 12.3897 (12)
		β = 103.8223(12)	*β* = 103.9526(26)
		V = 2530.5(3)	*V* = 2539.5(7)
$[Fe(acac)_3][Mn(hfac)_2]$ (**4**)	*P*-1	*a* = 9.4689(8)	*a* = 9.4640(6)
		b = 11.8310(6)	*b* = 11.8255(8)
		c = 16.5156(4)	*c* = 16.5080(11)
		α = 102.01(2)	*α* = 102.0220(18)
		β = 97.87(2)	*β* = 97.8590(18)
		γ = 112.67(7)	*γ* = 112.6820(17)
		V = 1620.7(1)	*V* = 1618.30(18)
$[Ni(hfac)_2][Fe(acac)_3][Ni(hfac)_2]$ (**5**)	$P2_1$	*a* =9.7712(1)	*a* = 9.7682(7)
		b = 20.7697(9)	*b* = 20.7583(15)
		c = 12.0639(1)	*c* = 12.0575(9)
		β = 103.11(4)	*β* = 103.1260(17)
		V = 2384.5(6)	*V* = 2381.0(3)

[a]at 20 °C.

Crystal Growth

Crystals of heterometallic diketonates suitable for X-ray structural measurements were obtained directly from the reaction procedures described above: $[Fe(acac)_3][Fe(hfac)_2]$ (**1**), 85 ^oC, 4 days; $[Fe(hfac)_2][Fe(acac)_3][Fe(hfac)_2]$ (**2** and **2a**), 85 ^oC, 6 days; $[Fe(hfac)_2][Fe(acac)_2(hfac)][Fe(hfac)_2]$ (**3**), 75 ^oC, 3 days; $[Fe(acac)_3][Mn(hfac)_2]$ (**4**), 90 ^oC, 5 days; $[Ni(hfac)_2][Fe(acac)_3][Ni(hfac)_2]$ (**5**), 95 ^oC, 5 days.

X-ray Crystallographic Procedures

The single crystal diffraction data for $[Fe(acac)_3][Fe(hfac)_2]$ (**1**) and $[Fe(hfac)_2][Fe(acac)_2(hfac)][Fe(hfac)_2]$ (**3**) were measured on a Bruker SMART APEX CCD-based X-ray diffractometer system equipped with a Mo-target X-ray tube (λ = 0.71073 Å). The single crystal diffraction data for two enantiomers of $[Fe(hfac)_2][Fe(acac)_3][Fe(hfac)_2]$ (**2** and **2a**), $[Fe(acac)_3][Mn(hfac)_2]$ (**4**) and $[Ni(hfac)_2][Fe(acac)_3][Ni(hfac)_2]$ (**5**) were collected on a Bruker D8 VENTURE with PHOTON 100 CMOS detector system using Mo radiation (λ = 0.71073 Å). Data reduction and integration were performed with the Bruker software package SAINT. Data were corrected for absorption effects using the empirical methods as implemented in SADABS. The structures were solved and refined by full-matrix least-squares procedures using the Bruker SHELXTL (version 6.14) software package. All non-hydrogen atoms were refined anisotropically and hydrogen atoms were included in idealized positions for structure factor calculations. Two of the CF_3 groups in **1**, **4**, and **5**, three of the CF_3 groups in **2a,** four of the CF_3 groups in **3**, and five of the CF_3 groups in **2** were found to be rotationally disordered. All fluorine atoms of the disordered parts were modeled with isotropic thermal parameters using similarity restraints. Some bond distances in the disordered moieties were also restrained. Crystallographic data for **1**-**5** and details of the data collection and structure refinement are listed in Tables S3 and S3a.

Table S3. Crystal Data and Structure Refinement Parameters for $[Fe(acac)_3][Fe(hfac)_2]$ (**1**), $[Fe(acac)_3][Mn(hfac)_2]$ (**4**), $[Fe(hfac)_2][Fe(acac)_3][Fe(hfac)_2]$ (**2**), and $[Ni(hfac)_2][Fe(acac)_3][Ni(hfac)_2]$ (**5**)

Compound	**1**	**4**	**2**	**5**
Empirical formula	$C_{25}H_{23}O_{10}F_{12}Fe_2$	$C_{25}H_{23}O_{10}F_{12}Mn$ Fe	$C_{35}H_{25}O_{14}F_{24}Fe_3$	$C_{35}H_{25}O_{14}F_{24}Ni_2Fe$
Formula weight	823.13	822.22	1293.10	1298.82
Temperature (K)	100(2)	100(2)	100(2)	100(2)
Wavelength (Å)	0.71073	0.71073	0.71073	0.71073
Crystal system	Triclinic	Triclinic	Monoclinic	Monoclinic
Space group	P-1	P-1	$P2_1$	$P2_1$
a (Å)	9.507(2)	9.4640(6)	9.7834(7)	9.7682(7)
b (Å)	11.729(3)	11.8255(8)	20.8414(16)	20.7583(15)
c (Å)	16.605(4)	16.5080(11)	12.0955(9)	12.0575(9)
α (°)	102.703(2)	102.0220(18)	90.00	90.00
β (°)	96.771(2)	97.8590(18)	102.5700(19)	103.1260(17)
γ (°)	112.595(2)	112.6820(17)	90.00	90.00
V (Å^3)	1625.0(7)	1618.30(18)	2407.2(3)	2381.0(3)
Z	2	2	2	2
ρ_{calcd} (g·cm^{-3})	1.682	1.687	1.784	1.812
μ (mm^{-1})	1.012	0.958	1.045	1.238
F(000)	826	824	1282	1290
Crystal size (mm)	0.25×0.10×0.10	0.20×0.18×0.17	0.37×0.22×0.12	0.18×0.17×0.14
θ range for data collection (°)	2.36–27.89	2.99–28.75	2.89–28.32	2.90–27.88
Reflections collected	13069	48869	45822	60549
Independent reflections	7052 [R_{int} = 0.0255]	8376 [R_{int} = 0.0431]	11964 [R_{int} = 0.0260]	11379 [R_{int} = 0.0388]
Transmission factors (min/max)	0.7859/0.9055	0.8314/0.8540	0.6984/0.8848	0.8079/0.8458
Data/restraints/params.	7052/36/466	8376/36/466	11964/91/736	11379/37/709
Flack x parameter			0.044(8)	0.005(11)
$R1$,[a] $wR2$[b] ($I > 2\sigma(I)$)	0.0481, 0.1106	0.0444, 0.0987	0.0291, 0.0656	0.0349, 0.0657
$R1$,[a] $wR2$[b] (all data)	0.0728, 0.1249	0.0729, 0.1110	0.0329, 0.0675	0.0450, 0.0698
Quality-of-fit[c]	1.037	1.016	1.026	1.061

[a] $R1 = \Sigma||F_o|-|F_c||/\Sigma|F_o|$. [b] $wR2 = [\Sigma[w(F_o^2-F_c^2)^2]/\Sigma[w(F_o^2)^2]]$.
[c] Quality-of-fit = $[\Sigma[w(F_o^2-F_c^2)^2]/(N_{obs}-N_{params})]^{½}$, based on all data.

Table S3a. Crystal Data and Structure Refinement Parameters for *Λ,Λ,Λ*-[Fe(hfac)$_2$][Fe(acac)$_3$][Fe(hfac)$_2$] (**2**), *Δ,Δ,Δ*-[Fe(hfac)$_2$][Fe(acac)$_3$][Fe(hfac)$_2$] (**2a**), and [Fe(hfac)$_2$][Fe(acac)$_2$(hfac)][Fe(hfac)$_2$] (**3**)

Compound	**2**	**2a**	**3**
Empirical formula	$C_{35}H_{25}O_{14}F_{24}Fe_3$	$C_{35}H_{25}O_{14}F_{24}Fe_3$	$C_{35}H_{19}O_{14}F_{30}Fe_3$
Formula weight	1293.10	1293.10	1401.05
Temperature (K)	100(2)	100(2)	100(2)
Wavelength (Å)	0.71073	0.71073	0.71073
Crystal system	Monoclinic	Monoclinic	Monoclinic
Space group	$P2_1$	$P2_1$	$C2/c$
a (Å)	9.7834(7)	9.7979(13)	20.934(3)
b (Å)	20.8414(16)	20.819(3)	13.481(2)
c (Å)	12.0955(9)	12.0998(16)	17.739(3)
α (°)	90.00	90.00	90.00
β (°)	102.5700(19)	102.659(3)	92.848(2)
γ (°)	90.00	90.00	90.00
V (Å^3)	2407.2(3)	2408.2(6)	4999.8(14)
Z	2	2	4
ρ_{calcd} (g·cm^{-3})	1.784	1.783	1.861
μ (mm^{-1})	1.045	1.045	1.031
F(000)	1282	1282	2756
Crystal size (mm)	0.37×0.22×0.12	0.32×0.13×0.10	0.30×0.25×0.16
θ range for data collection (°)	2.89–28.32	2.89–28.29	2.16–28.23
Reflections collected	45822	56462	21346
Independent reflections	11964 [R_{int} = 0.0260]	11929 [R_{int} = 0.0250]	5851 [R_{int} = 0.0226]
Transmission factors (min/max)	0.6984/0.8848	0.7310/0.9027	0.7473/0.8524
Data/restraints/params.	11964/91/736	11929/55/718	5851/36/391
Flack x parameter	0.044(8)	0.007(7)	
$R1$,[a] $wR2$[b] ($I > 2\sigma(I)$)	0.0291, 0.0656	0.0293, 0.0687	0.0422, 0.1087
$R1$,[a] $wR2$[b] (all data)	0.0329, 0.0675	0.0348, 0.0718	0.0494, 0.1149
Quality-of-fit[c]	1.026	1.005	1.034

[a] $R1 = \Sigma||F_o|-|F_c||/\Sigma|F_o|$. [b] $wR2 = [\Sigma[w(F_o^2-F_c^2)^2]/\Sigma[w(F_o^2)^2]]$

[c] Quality-of-fit = $[\Sigma[w(F_o^2-F_c^2)^2]/(N_{obs}-N_{params})]^{1/2}$, based on all data.

Molecular Structures of Homo- and Heterometallic Diketonates 1-5

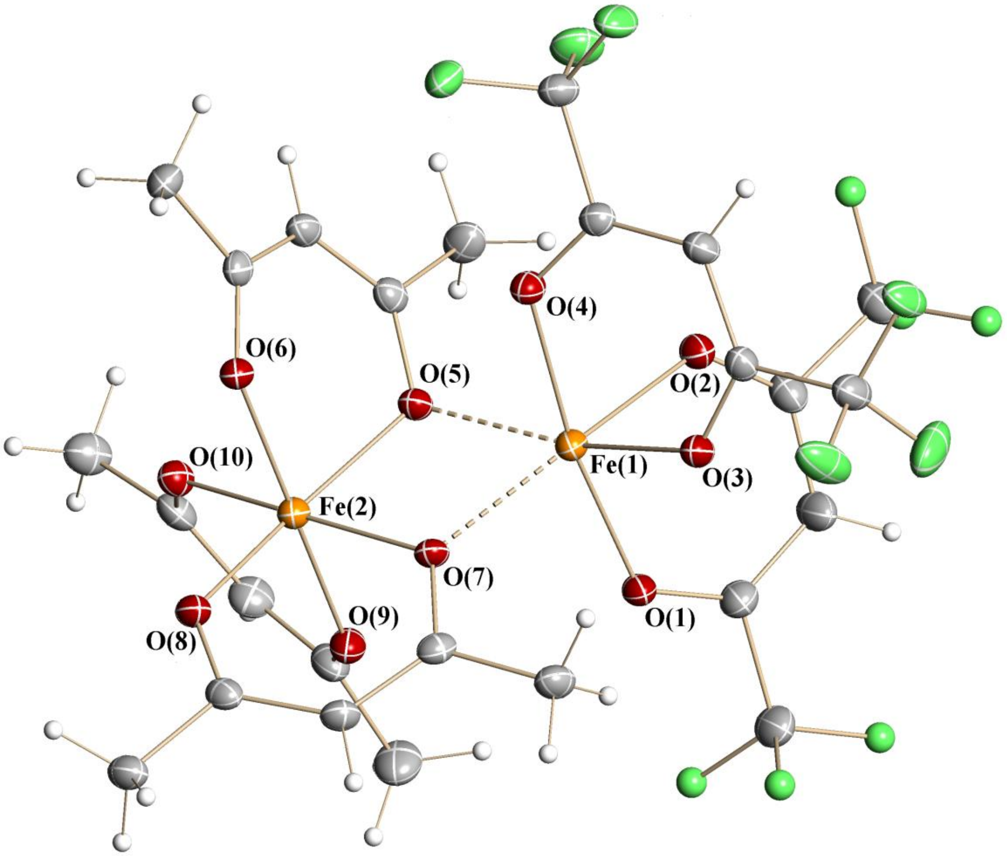

Figure S5. Solid state structure of homometallic diketonate $[Fe^{III}(acac)_3][Fe^{II}(hfac)_2]$ (**1**). Atoms are represented by thermal ellipsoids at the 40% probability level. Hydrogen and some of the fluorine atoms are represented by spheres of arbitrary radii. Only one orientation of disordered CF_3 groups is shown. Only iron and oxygen atoms are labeled. Bridging Fe–O interactions are drawn by dashed lines.

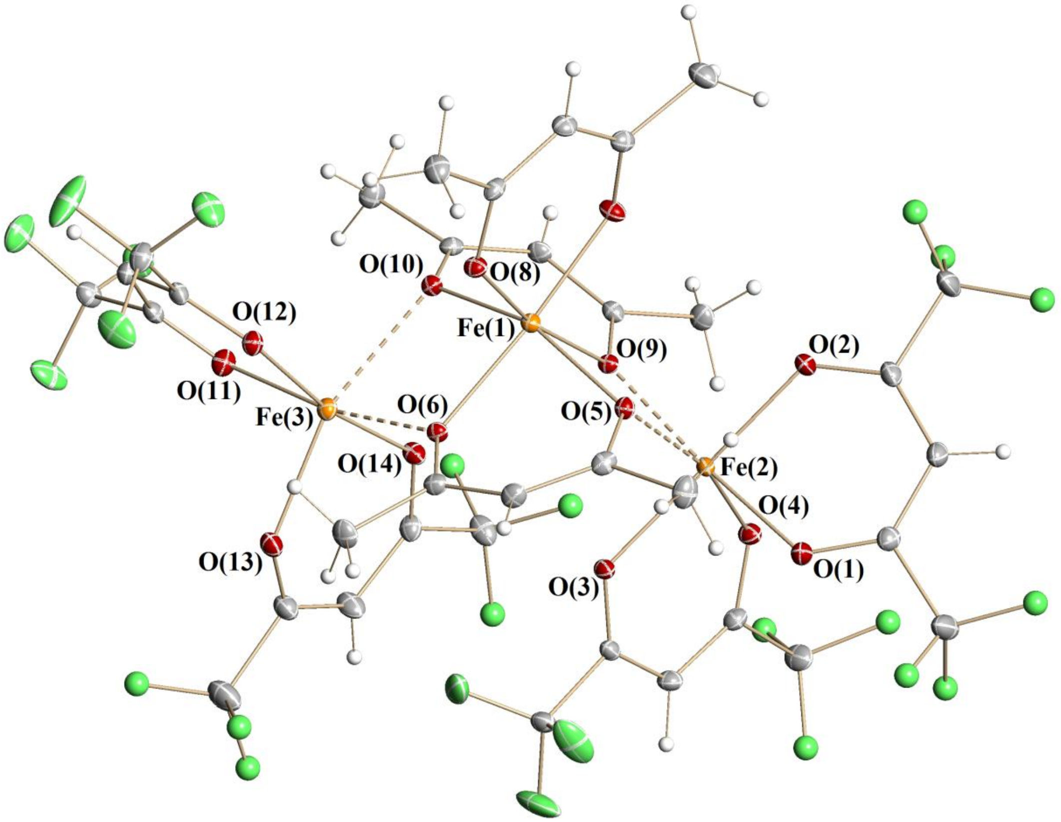

Figure S6. Solid state structure of *Λ,Λ,Λ*-enantiomer of homometallic diketonate $[Fe^{II}(hfac)_2][Fe^{III}(acac)_3][Fe^{II}(hfac)_2]$ (**2**). Atoms are represented by thermal ellipsoids at the 40% probability level. Hydrogen and some of the fluorine atoms are represented by spheres of arbitrary radii. Only one orientation of disordered CF_3 groups is shown. Only iron and oxygen atoms are labeled. Bridging Fe–O interactions are drawn by dashed lines.

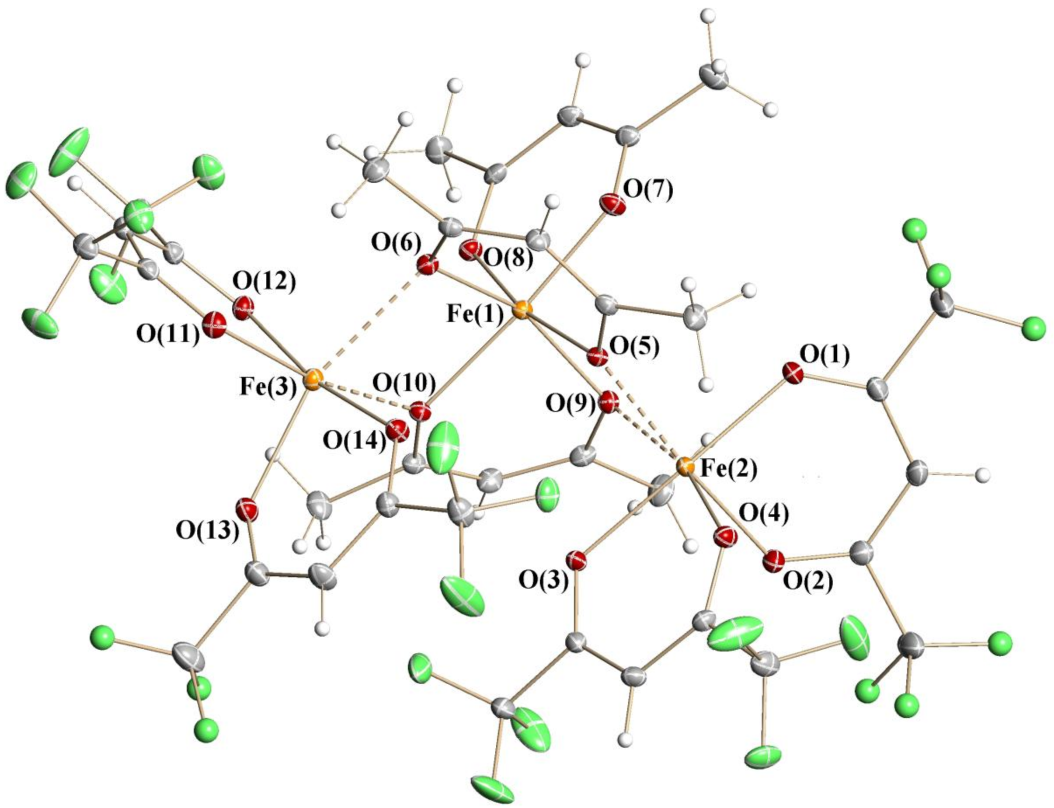

Figure S6a. Solid state structure of *Δ*,*Δ*,*Δ*-enantiomer of homometallic diketonate $[Fe^{II}(hfac)_2][Fe^{III}(acac)_3][Fe^{II}(hfac)_2]$ (**2a**). Atoms are represented by thermal ellipsoids at the 40% probability level. Hydrogen and some of the fluorine atoms are represented by spheres of arbitrary radii. Only one orientation of disordered CF_3 groups is shown. Only iron and oxygen atoms are labeled. Bridging Fe–O interactions are drawn by dashed lines.

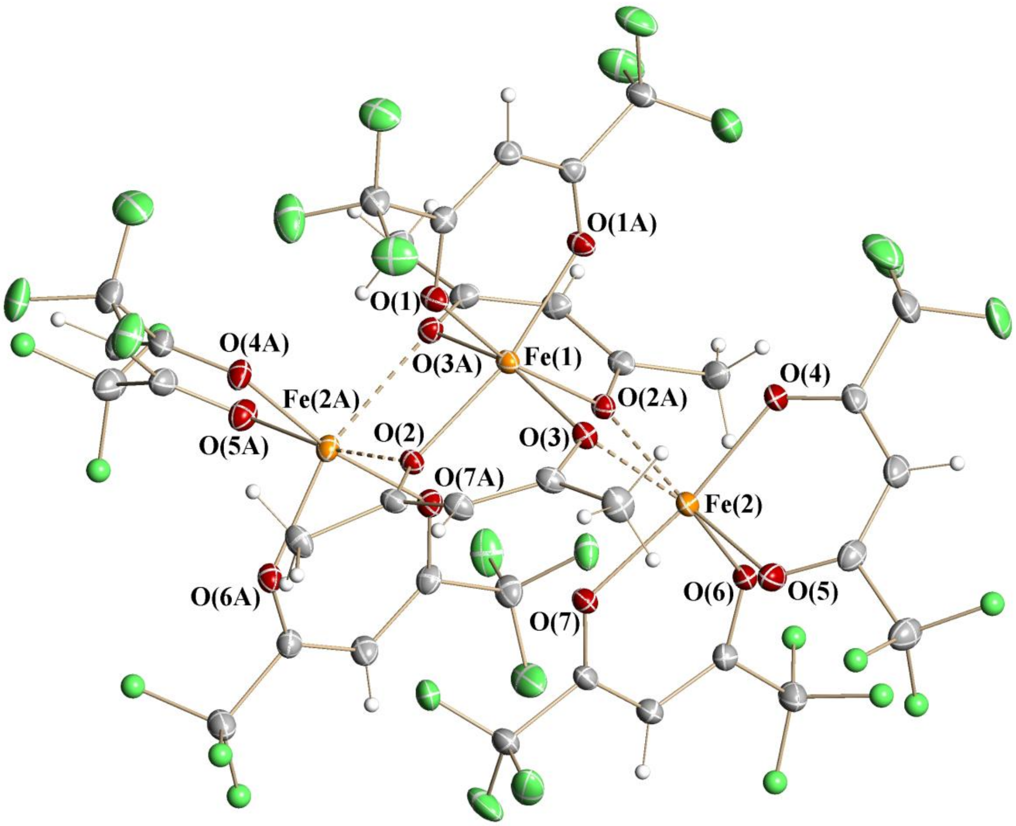

Figure S7. Solid state structure of homometallic diketonate $[Fe^{II}(hfac)_2][Fe^{III}(acac)_2(hfac)][Fe^{II}(hfac)_2]$ (**3**). Atoms are represented by thermal ellipsoids at the 40% probability level. Hydrogen and some of the fluorine atoms are represented by spheres of arbitrary radii. Only one orientation of disordered CF_3 groups is shown. Only iron and oxygen atoms are labeled. Bridging Fe–O interactions are drawn by dashed lines.

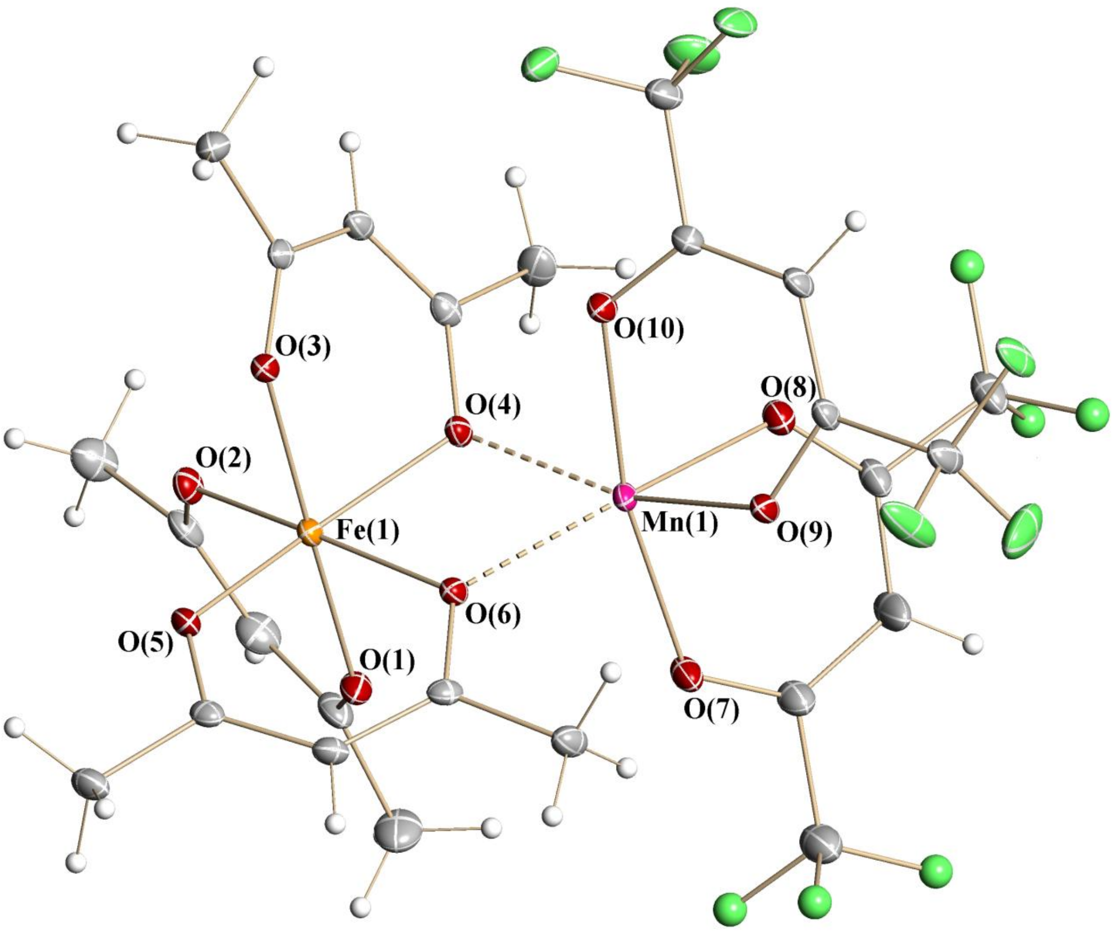

Figure S8. Solid state structure of heterometallic diketonate $[Fe^{III}(acac)_3][Mn^{II}(hfac)_2]$ (**4**). Atoms are represented by thermal ellipsoids at the 40% probability level. Hydrogen and some of the fluorine atoms are represented by spheres of arbitrary radii. Only one orientation of disordered CF_3 groups is shown. Only metal and oxygen atoms are labeled. Bridging Mn–O interactions are drawn by dashed lines.

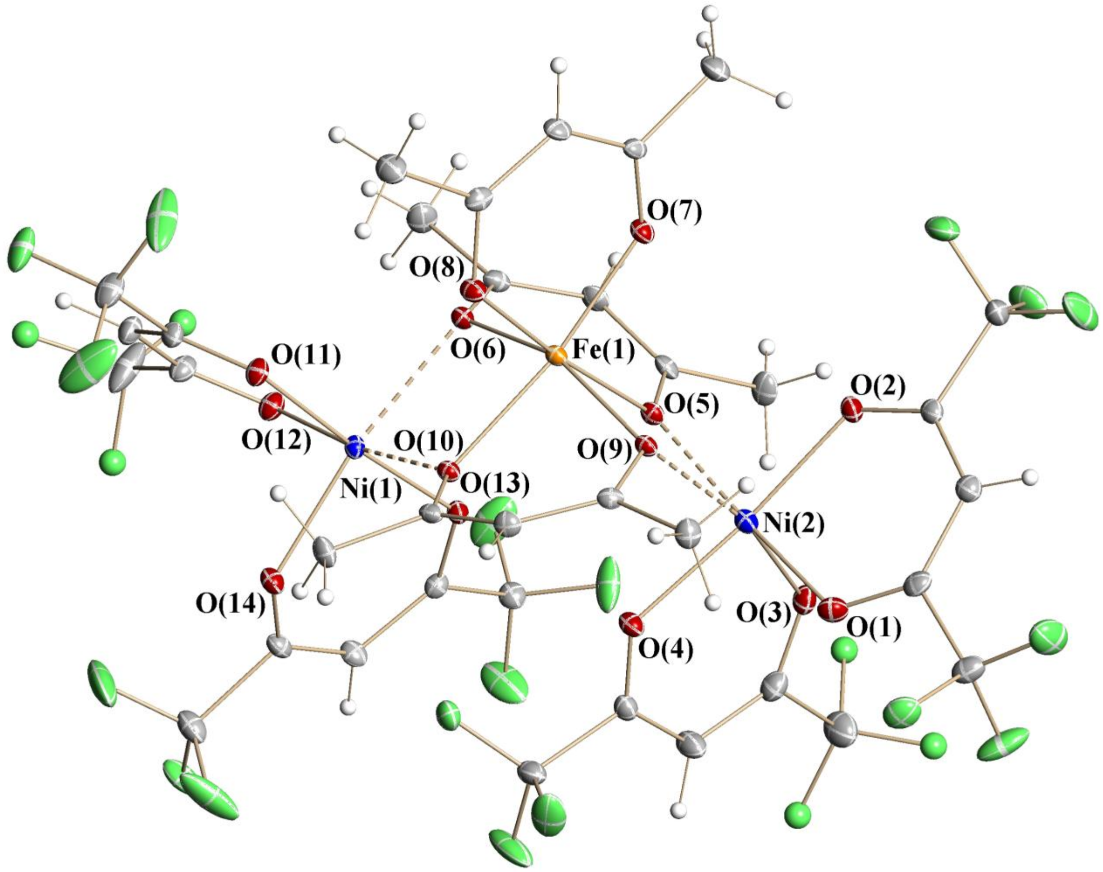

Figure S9. Solid state structure of heterometallic diketonate $[Ni^{II}(hfac)_2][Fe^{III}(acac)_3][Ni^{II}(hfac)_2]$ (**5**). Atoms are represented by thermal ellipsoids at the 40% probability level. Hydrogen and some of the fluorine atoms are represented by spheres of arbitrary radii. Only one orientation of disordered CF_3 groups is shown. Only metal and oxygen atoms are labeled. Bridging Ni–O interactions are drawn by dashed lines.

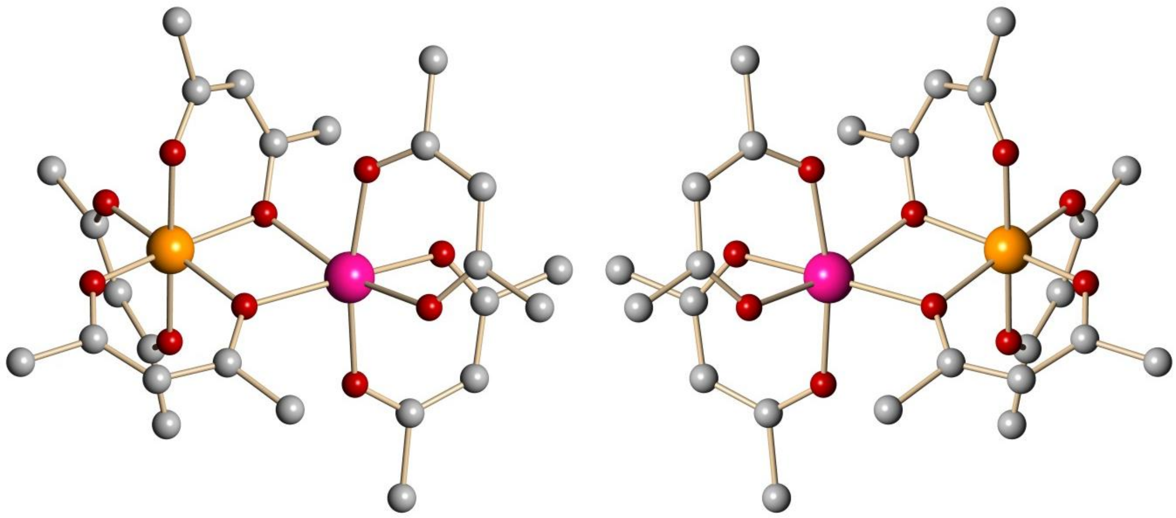

Figure S10. *Λ*,*Λ*- (left) and *Δ*,*Δ*- (right) enantiomers in the crystal structure of heterometallic diketonate $[Fe^{III}(acac)_3][Mn^{II}(hfac)_2]$ (**4**). Color scheme: iron, dark orange; manganese, deep pink; oxygen, red; carbon, gray. Atoms are represented by spheres of arbitrary radii. Hydrogen and fluorine atoms are omitted for clarity.

Table S4. Selected Bond Distances (Å) and Angles (deg.) in the Structure of $[Fe^{III}(acac)_3][Fe^{II}(hfac)_2]$ (**1**)

**Distances**		**Angles**		**Angles**	
Fe(1)−O(1)	2.039(2)	O(1)−Fe(1)−O(2)	85.32(9)	O(5)−Fe(2)−O(7)	80.77(9)
Fe(1)−O(2)	2.094(2)	O(1)−Fe(1)−O(3)	92.48(9)	O(6)−Fe(2)−O(5)	86.31(9)
Fe(1)−O(3)	2.106(2)	O(1)−Fe(1)−O(4)	171.30(9)	O(6)−Fe(2)−O(7)	96.40(8)
Fe(1)−O(4)	2.054(2)	O(1)−Fe(1)−O(5)	103.64(8)	O(6)−Fe(2)−O(8)	87.63(9)
Fe(1)−O(5)*	2.188(2)	O(1)−Fe(1)−O(7)	89.35(8)	O(6)−Fe(2)−O(9)	176.88(9)
Fe(1)−O(7)*	2.178(2)	O(2)−Fe(1)−O(3)	86.16(9)	O(6)−Fe(2)−O(10)	89.37(9)
		O(2)−Fe(1)−O(4)	86.80(9)	O(7)−Fe(2)−O(8)	88.36(9)
Fe(2)−O(6)	2.007(2)	O(2)−Fe(1)−O(5)	99.56(9)	O(7)−Fe(2)−O(9)	86.05(9)
Fe(2)−O(8)	1.964(2)	O(2)−Fe(1)−O(7)	170.72(9)	O(7)−Fe(2)−O(10)	172.11(9)
Fe(2)−O(9)	1.977(2)	O(3)−Fe(1)−O(4)	83.29(8)	O(8)−Fe(2)−O(5)	166.87(9)
Fe(2)−O(10)	1.976(2)	O(3)−Fe(1)−O(5)	163.23(8)	O(8)−Fe(2)−O(9)	94.39(10)
Fe(2)−O(5)"	2.015(2)	O(3)−Fe(1)−O(7)	101.67(9)	O(8)−Fe(2)−O(10)	97.28(9)
Fe(2)−O(7)"	2.055(2)	O(4)−Fe(1)−O(5)	81.32(8)	O(9)−Fe(2)−O(5)	92.18(9)
		O(4)−Fe(1)−O(7)	98.94(8)	O(9)−Fe(2)−O(10)	88.02(9)
		O(5)−Fe(1)−O(7)	74.32(8)	O(10)−Fe(2)−O(5)	94.29(9)

*- bridging oxygen; "- chelating-bridging oxygen

Table S5. Selected Bond Distances (Å) and Angles (deg.) in the Structure of *Λ,Λ,Λ*-$[Fe^{II}(hfac)_2][Fe^{III}(acac)_3][Fe^{II}(hfac)_2]$ (**2**)

**Distances**		**Angles**		**Angles**	
Fe(1)−O(7)	1.962(2)	O(5)−Fe(1)−O(6)	85.18(7)	O(1)−Fe(2)−O(2)	86.32(7)
Fe(1)−O(8)	1.954(2)	O(6)−Fe(1)−O(7)	173.97(7)	O(1)−Fe(2)−O(3)	91.14(7)
Fe(1)–O(5)"	2.005(2)	O(6)−Fe(1)−O(8)	86.63(7)	O(1)−Fe(2)−O(4)	85.44(7)
Fe(1)−O(6)"	2.047(2)	O(6)−Fe(1)−O(9)	103.49(6)	O(1)−Fe(2)−O(5)	101.25(7)
Fe(1)−O(9)"	2.047(2)	O(6)−Fe(1)−O(10)	80.61(7)	O(1)−Fe(2)−O(9)	172.83(7)
Fe(1)−O(10)"	2.004(2)	O(7)−Fe(1)−O(5)	96.90(7)	O(2)−Fe(2)−O(3)	173.98(8)
		O(7)−Fe(1)−O(8)	87.48(7)	O(2)−Fe(2)−O(4)	99.68(7)
Fe(2)−O(1)	2.065(2)	O(7)−Fe(1)−O(9)	82.46(7)	O(2)−Fe(2)−O(5)	92.46(7)
Fe(2)−O(2)	2.020(2)	O(7)−Fe(1)−O(10)	99.12(7)	O(2)−Fe(2)−O(9)	89.23(7)
Fe(2)−O(3)	2.034(2)	O(8)−Fe(1)−O(5)	97.78(7)	O(3)−Fe(2)−O(4)	85.54(7)
Fe(2)−O(4)	2.083(2)	O(8)−Fe(1)−O(9)	169.49(7)	O(3)−Fe(2)−O(5)	82.68(6)
Fe(2)−O(5)*	2.235(2)	O(8)−Fe(1)−O(10)	99.58(7)	O(3)−Fe(2)−O(9)	92.74(7)
Fe(2)−O(9)*	2.149(2)	O(9)−Fe(1)−O(5)	80.49(6)	O(4)−Fe(2)−O(5)	166.53(7)
		O(9)−Fe(1)−O(10)	85.08(6)	O(4)−Fe(2)−O(9)	100.87(6)
Fe(3)−O(11)	2.057(2)	O(10)−Fe(1)−O(5)	156.79(7)	O(5)−Fe(2)−O(9)	73.30(6)
Fe(3)−O(12)	2.017(2)			O(10)−Fe(3)−O(6)	71.40(6)
Fe(3)−O(13)	2.070(2)			O(11)−Fe(3)−O(6)	169.94(7)
Fe(3)−O(14)	2.026(2)			O(11)−Fe(3)−O(10)	101.10(7)
Fe(3)−O(6)*	2.196(2)			O(11)−Fe(3)−O(12)	86.24(7)
Fe(3)−O(10)*	2.293(2)			O(11)−Fe(3)−O(13)	89.97(7)
				O(11)−Fe(3)−O(14)	93.97(7)
				O(12)−Fe(3)−O(6)	86.73(6)
				O(12)−Fe(3)−O(10)	90.79(6)
				O(12)−Fe(3)−O(13)	104.90(7)
				O(12)−Fe(3)−O(14)	169.93(7)
				O(13)−Fe(3)−O(6)	99.20(7)
				O(13)−Fe(3)−O(10)	161.44(7)
				O(13)−Fe(3)−O(14)	85.17(7)
				O(14)−Fe(3)−O(6)	91.63(7)
				O(14)−Fe(3)−O(10)	79.28(6)

*- bridging oxygen; "- chelating-bridging oxygen

Table S5a. Selected Bond Distances (Å) and Angles (deg.) in the Structure of *Δ,Δ,Δ*-[Fe^{II}(hfac)$_2$][Fe^{III}(acac)$_3$][Fe^{II}(hfac)$_2$] (**2a**)

**Distances**		**Angles**		**Angles**	
Fe(1)−O(7)	1.962(2)	O(5)−Fe(1)−O(6)	84.98(6)	O(1)−Fe(2)−O(2)	86.35(7)
Fe(1)−O(8)	1.954(2)	O(6)−Fe(1)−O(7)	99.10(7)	O(1)−Fe(2)−O(3)	173.95(7)
Fe(1)–O(5)"	2.053(2)	O(6)−Fe(1)−O(8)	99.71(7)	O(1)−Fe(2)−O(4)	99.53(7)
Fe(1)−O(6)"	2.004(2)	O(6)−Fe(1)−O(9)	156.82(7)	O(1)−Fe(2)−O(5)	89.24(7)
Fe(1)−O(9)"	2.001(2)	O(6)−Fe(1)−O(10)	80.60(6)	O(1)−Fe(2)−O(9)	92.50(7)
Fe(1)−O(10)"	2.051(2)	O(7)−Fe(1)−O(5)	82.39(7)	O(2)−Fe(2)−O(3)	91.06(7)
		O(7)−Fe(1)−O(8)	87.52(7)	O(2)−Fe(2)−O(4)	85.46(7)
Fe(2)−O(1)	2.019(2)	O(7)−Fe(1)−O(9)	96.88(7)	O(2)−Fe(2)−O(5)	172.95(7)
Fe(2)−O(2)	2.063(2)	O(7)−Fe(1)−O(10)	173.91(7)	O(2)−Fe(2)−O(9)	101.23(7)
Fe(2)−O(3)	2.035(2)	O(8)−Fe(1)−O(5)	169.46(7)	O(3)−Fe(2)−O(4)	85.70(7)
Fe(2)−O(4)	2.082(2)	O(8)−Fe(1)−O(9)	97.64(7)	O(3)−Fe(2)−O(5)	92.79(7)
Fe(2)−O(5)*	2.148(2)	O(8)−Fe(1)−O(10)	86.53(7)	O(3)−Fe(2)−O(9)	82.63(6)
Fe(2)−O(9)*	2.237(2)	O(9)−Fe(1)−O(5)	80.61(6)	O(4)−Fe(2)−O(5)	100.71(6)
		O(9)−Fe(1)−O(10)	85.25(7)	O(4)−Fe(2)−O(9)	166.62(6)
Fe(3)−O(11)	2.057(2)	O(10)−Fe(1)−O(5)	103.61(6)	O(5)−Fe(2)−O(9)	73.44(6)
Fe(3)−O(12)	2.021(2)			O(10)−Fe(3)−O(6)	71.52(6)
Fe(3)−O(13)	2.068(2)			O(11)−Fe(3)−O(6)	101.06(6)
Fe(3)−O(14)	2.031(2)			O(11)−Fe(3)−O(10)	169.59(7)
Fe(3)−O(6)*	2.292(2)			O(11)−Fe(3)−O(12)	86.26(7)
Fe(3)−O(10)*	2.195(2)			O(11)−Fe(3)−O(13)	90.04(7)
				O(11)−Fe(3)−O(14)	93.91(7)
				O(12)−Fe(3)−O(6)	90.82(6)
				O(12)−Fe(3)−O(10)	86.57(6)
				O(12)−Fe(3)−O(13)	104.83(7)
				O(12)−Fe(3)−O(14)	169.98(7)
				O(13)−Fe(3)−O(6)	161.44(6)
				O(13)−Fe(3)−O(10)	99.08(6)
				O(13)−Fe(3)−O(14)	85.19(7)
				O(14)−Fe(3)−O(6)	79.30(6)
				O(14)−Fe(3)−O(10)	91.84(7)

*- bridging oxygen; "-chelating-bridging oxygen

Table S6. Selected Bond Distances (Å) and Angles (deg.) in the Structure of $[Fe^{II}(hfac)_2][Fe^{III}(acac)_2(hfac)][Fe^{II}(hfac)_2]$ (**3**)

**Distances**		**Angles**		**Angles**	
Fe(1)−O(1)	2.008(2)	O(1)−Fe(1)−O(1A)	84.59(10)	O(3)−Fe(2)−O(2A)	71.75(6)
Fe(1)−O(2)"	2.012(2)	O(1)−Fe(1)−O(2)	84.15(7)	O(4)−Fe(2)−O(2A)	85.69(7)
Fe(1)–O(3)"	1.986(2)	O(1)−Fe(1)−O(2A)	167.50(7)	O(4)−Fe(2)−O(3)	90.14(7)
		O(1)−Fe(1)−O(3)	94.84(7)	O(4)−Fe(2)−O(5)	86.58(7)
Fe(2)−O(4)	2.019(2)	O(1)−Fe(1)−O(3A)	99.92(7)	O(4)−Fe(2)−O(6)	103.34(7)
Fe(2)−O(5)	2.061(2)	O(1A)−Fe(1)−O(2A)	84.15(7)	O(4)−Fe(2)−O(7)	170.45(7)
Fe(2)−O(6)	2.053(2)	O(2)−Fe(1)−O(1A)	167.50(7)	O(5)−Fe(2)−O(2A)	170.77(7)
Fe(2)−O(7)	2.035(2)	O(2)−Fe(1)−O(2A)	107.54(9)	O(5)−Fe(2)−O(3)	103.26(7)
Fe(2)−O(2A)*	2.201(2)	O(2)−Fe(1)−O(3)	86.45(7)	O(5)−Fe(2)−O(6)	88.02(7)
Fe(2)−O(3)*	2.264(2)	O(2)−Fe(1)−O(3A)	81.77(7)	O(5)−Fe(2)−O(7)	94.80(7)
		O(3)−Fe(1)−O(1A)	99.92(7)	O(6)−Fe(2)−O(2A)	98.65(6)
		O(3)−Fe(1)−O(2A)	81.77(7)	O(6)−Fe(2)−O(3)	163.02(6)
		O(3)−Fe(1)−O(3A)	160.02(10)	O(6)−Fe(2)−O(7)	86.16(7)
		O(3A)−Fe(1)−O(1A)	94.84(7)	O(7)−Fe(2)−O(2A)	92.02(6)
		O(3A)−Fe(1)−O(2A)	86.45(7)	O(7)−Fe(2)−O(3)	80.35(7)

*- bridging oxygen; "-chelating-bridging oxygen

Table S7. Selected Bond Distances (Å) and Angles (deg.) in the Structure of $[Fe^{III}(acac)_3][Mn^{II}(hfac)_2]$ (**4**)

**Distances**		**Angles**		**Angles**	
Fe(1)−O(1)	1.971(2)	O(1)−Fe(1)−O(2)	88.05(8)	O(6)−Mn(1)−O(4)	73.98(6)
Fe(1)−O(2)	1.971(2)	O(1)−Fe(1)−O(3)	176.70(8)	O(7)−Mn(1)−O(4)	106.83(7)
Fe(1)−O(3)	2.001(2)	O(1)−Fe(1)−O(4)	91.73(7)	O(7)−Mn(1)−O(6)	90.57(7)
Fe(1)−O(5)	1.964(2)	O(1)−Fe(1)−O(5)	94.90(8)	O(7)−Mn(1)−O(8)	83.93(7)
Fe(1)−O(4)"	2.014(2)	O(1)−Fe(1)−O(6)	85.25(7)	O(7)−Mn(1)−O(9)	91.98(7)
Fe(1)−O(6)"	2.060(2)	O(2)−Fe(1)−O(3)	89.81(7)	O(7)−Mn(1)−O(10)	168.48(7)
		O(2)−Fe(1)−O(4)	94.45(7)	O(8)−Mn(1)−O(4)	99.16(7)
Mn(1)−O(7)	2.095(2)	O(2)−Fe(1)−O(5)	97.37(7)	O(8)−Mn(1)−O(6)	169.62(7)
Mn(1)−O(8)	2.138(2)	O(2)−Fe(1)−O(6)	171.90(7)	O(8)−Mn(1)−O(9)	86.00(7)
Mn(1)−O(9)	2.158(2)	O(3)−Fe(1)−O(4)	85.92(7)	O(9)−Mn(1)−O(4)	160.87(7)
Mn(1)−O(10)	2.112(2)	O(3)−Fe(1)−O(5)	87.88(7)	O(9)−Mn(1)−O(6)	103.03(7)
Mn(1)−O(4)*	2.212(2)	O(3)−Fe(1)−O(6)	96.67(7)	O(10)−Mn(1)−O(4)	80.45(6)
Mn(1)−O(6)*	2.195(2)	O(4)−Fe(1)−O(5)	166.63(7)	O(10)−Mn(1)−O(6)	100.15(7)
		O(4)−Fe(1)−O(6)	81.23(7)	O(10)−Mn(1)−O(8)	86.11(7)
		O(5)−Fe(1)−O(6)	87.75(7)	O(10)−Mn(1)−O(9)	81.55(7)

*- bridging oxygen; "-chelating-bridging oxygen

Table S8. Selected Bond Distances (Å) and Angles (deg.) in the Structure of $[Ni^{II}(hfac)_2][Fe^{III}(acac)_3][Ni^{II}(hfac)_2]$ (**5**)

**Distances**		**Angles**		**Angles**	
Fe(1)−O(7)	1.956(2)	O(5)−Fe(1)−O(10)	101.79(8)	O(10)−Ni(1)−O(6)	75.61(8)
Fe(1)−O(8)	1.961(2)	O(6)−Fe(1)−O(5)	85.49(8)	O(11)−Ni(1)−O(6)	92.24(9)
Fe(1)–O(5)"	2.047(2)	O(6)−Fe(1)−O(7)	97.97(9)	O(11)−Ni(1)−O(10)	88.11(8)
Fe(1)−O(6)"	2.003(2)	O(6)−Fe(1)−O(8)	96.29(9)	O(11)−Ni(1)−O(12)	90.63(9)
Fe(1)−O(9)"	2.002(2)	O(6)−Fe(1)−O(9)	157.44(8)	O(11)−Ni(1)−O(13)	175.56(10)
Fe(1)−O(10)"	2.053(2)	O(6)−Fe(1)−O(10)	80.30(8)	O(11)−Ni(1)−O(14)	94.91(9)
		O(7)−Fe(1)−O(5)	87.22(9)	O(12)−Ni(1)−O(6)	97.58(9)
Ni(1)−O(11)	1.984(2)	O(7)−Fe(1)−O(8)	87.84(9)	O(12)−Ni(1)−O(10)	173.02(9)
Ni(1)−O(12)	2.012(2)	O(7)−Fe(1)−O(9)	98.90(9)	O(12)−Ni(1)−O(13)	88.34(9)
Ni(1)–O(13)	2.003(2)	O(7)−Fe(1)−O(10)	170.61(9)	O(12)−Ni(1)−O(14)	86.60(9)
Ni(1)−O(14)	2.035(2)	O(8)−Fe(1)−O(5)	174.94(9)	O(13)−Ni(1)−O(6)	83.61(8)
Ni(1)−O(6)*	2.178(2)	O(8)−Fe(1)−O(9)	99.21(9)	O(13)−Ni(1)−O(10)	92.40(8)
Ni(1)−O(10)*	2.088(2)	O(8)−Fe(1)−O(10)	83.20(9)	O(13)−Ni(1)−O(14)	89.34(9)
		O(9)−Fe(1)−O(5)	80.49(8)	O(14)−Ni(1)−O(6)	171.67(9)
Ni(2)−O(1)	2.015(2)	O(9)−Fe(1)−O(10)	85.34(8)	O(14)−Ni(1)−O(10)	100.34(8)
Ni(2)−O(2)	1.995(2)			O(1)−Ni(2)−O(2)	89.84(9)
Ni(2)−O(3)	2.031(2)			O(1)−Ni(2)−O(3)	89.12(9)
Ni(2)−O(4)	1.998(2)			O(1)−Ni(2)−O(4)	90.53(9)
Ni(2)−O(5)*	2.136(2)			O(1)−Ni(2)−O(5)	172.03(9)
Ni(2)−O(9)*	2.222(2)			O(1)−Ni(2)−O(9)	99.22(9)
				O(2)−Ni(2)−O(3)	98.79(9)
				O(2)−Ni(2)−O(4)	172.63(9)
				O(2)−Ni(2)−O(5)	86.70(8)
				O(2)−Ni(2)−O(9)	92.36(8)
				O(3)−Ni(2)−O(4)	88.57(9)
				O(3)−Ni(2)−O(5)	98.50(8)
				O(3)−Ni(2)−O(9)	166.11(8)
				O(4)−Ni(2)−O(5)	91.99(9)
				O(4)−Ni(2)−O(9)	80.32(8)
				O(5)−Ni(2)−O(9)	73.77(8)

*- bridging oxygen; "-chelating-bridging oxygen

Theoretical Calculations

All geometry optimizations were performed at the DFT level of theory using hybrid exchange-correlation functional PBE0. All atoms were described by the def2-TZVP basis sets. The nature of stationary points on the potential energy surface (PES) was determined by calculating the full Hessian matrix followed by computing harmonic vibrational frequencies. All structures reported were found to be true minima on the corresponding PES (no imaginary frequencies). All calculations were carried out utilizing the Firefly program package.

Spin states of systems under investigations were taken as:

System	*S*
$[Fe^{III}(acac)_3][Mn^{II}(hfac)_2]$ (**4**)	10/2
$[Mn^{III}(acac)_3][Fe^{II}(hfac)_2]$ (**4´**)	8/2
$[Fe^{III}(hfac)_3][Mn^{II}(acac)_2]$	10/2
$[Mn^{III}(hfac)_3][Fe^{II}(acac)_2]$	8/2

In order to obtain a better evaluation of energetics, single-point calculations were performed utilizing the recently developed double-hybrid DFT functional with empiric dispersion corrections (here B2PLYP-D3). All atoms were described by all-electron relativistically re-contracted triple-ζ basis sets (TZVP). Relativistic effects were accounted through the ZORA approach. All calculations at this level of theory were executed using ORCA (v. 3.0.1) program suite.

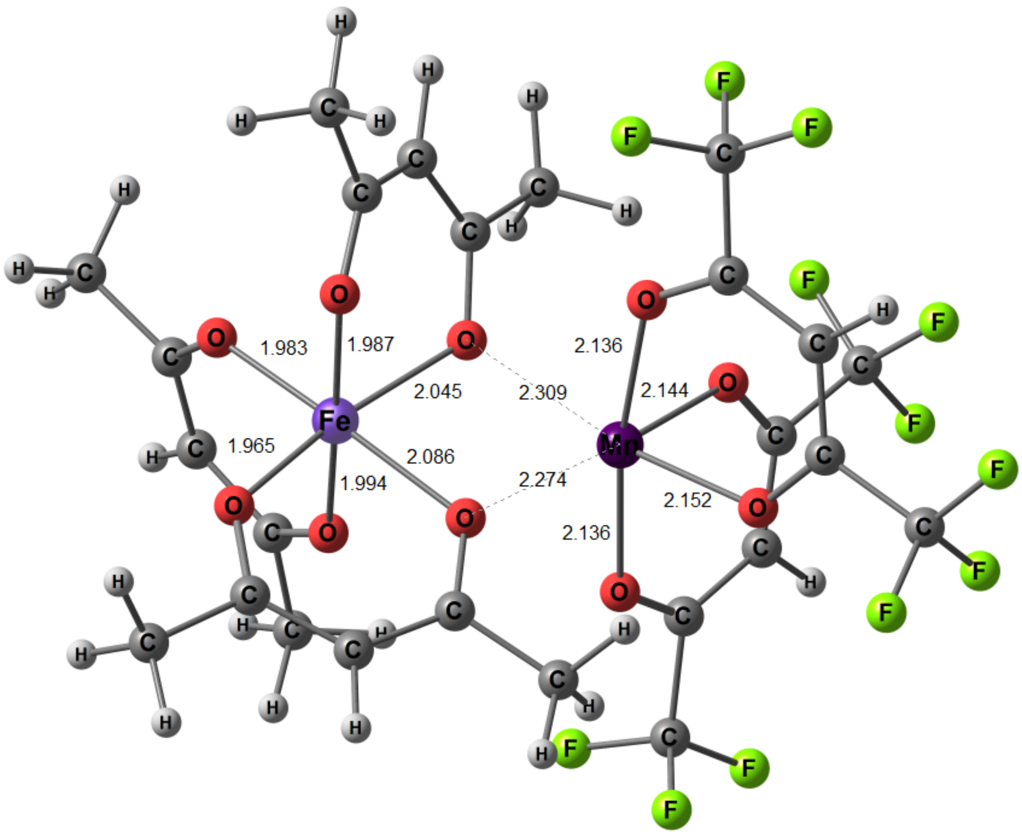

Figure S11. Equilibrium geometry configuration of heterobimetallic complex $[Fe^{III}(acac)_3][Mn^{II}(hfac)_2]$ (**4**) optimized at the PBE0/def2-TZVP level of theory.

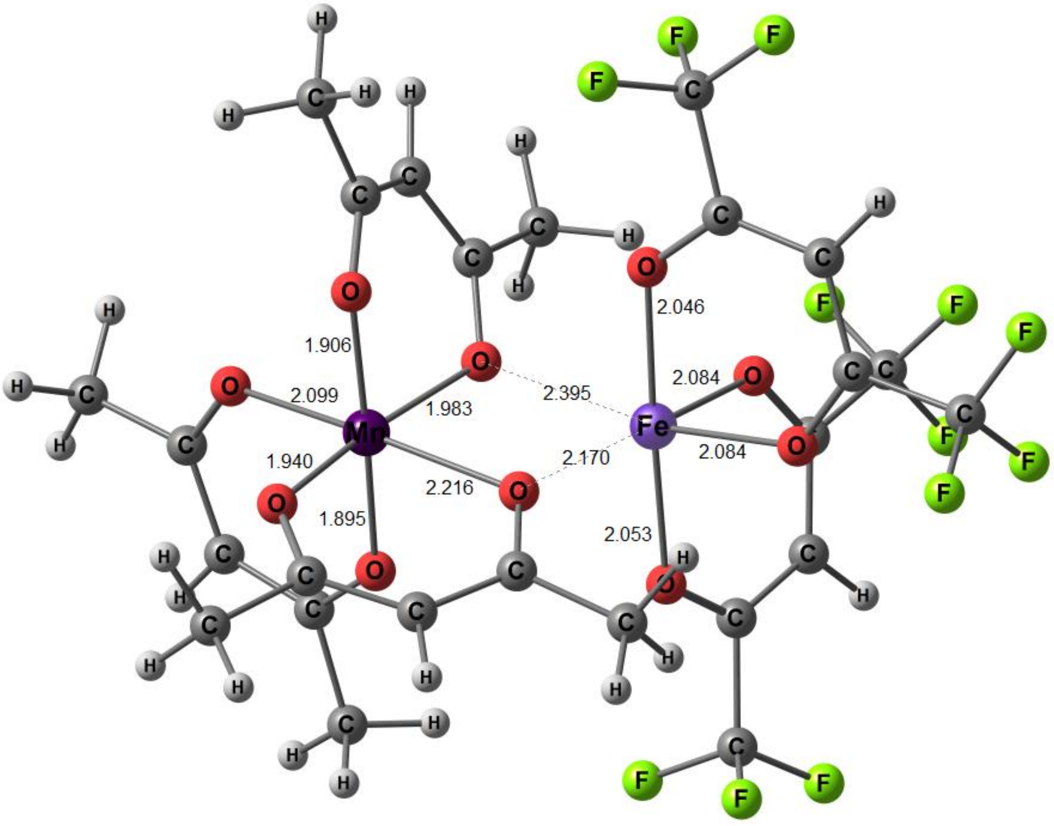

Figure S12. Equilibrium geometry configuration of heterobimetallic complex $[Mn^{III}(acac)_3][Fe^{II}(hfac)_2]$ (**4´**) optimized at the PBE0/def2-TZVP level of theory.

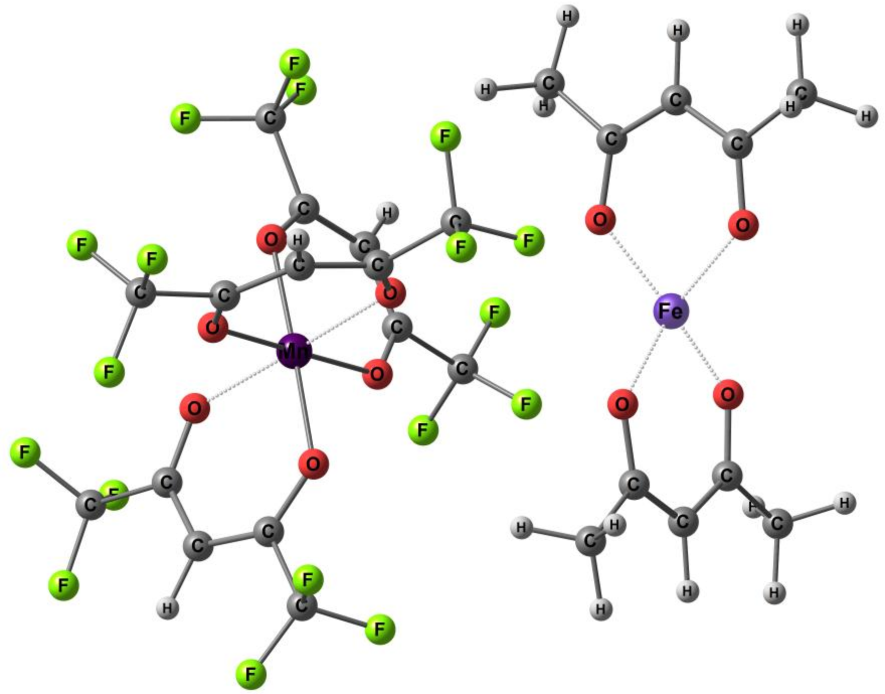

Figure S13. Equilibrium configuration of geometry optimization for $[Mn^{III}(hfac)_3][Fe^{II}(acac)_2]$ system at the PBE0/def2-TZVP level of theory.

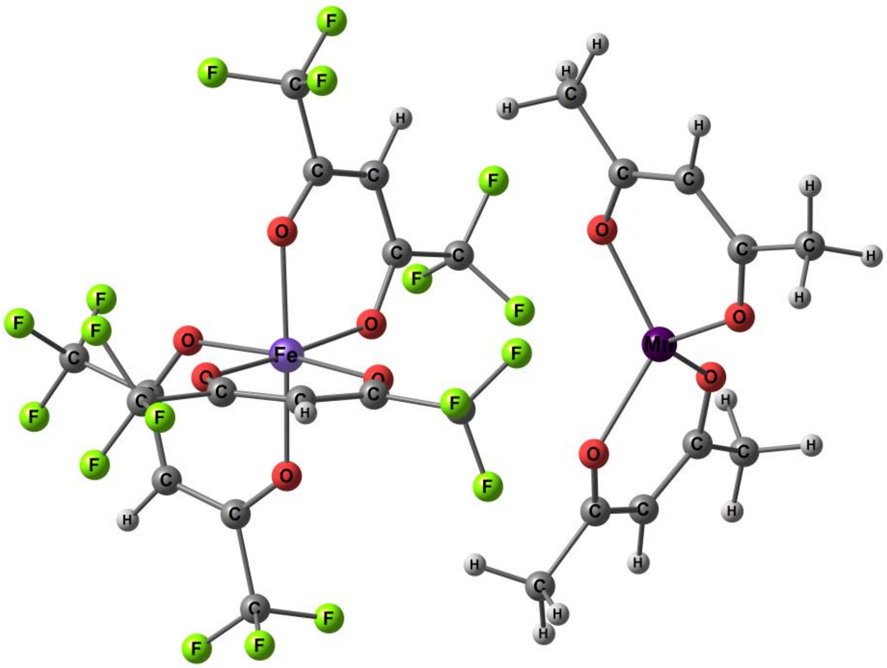

Figure S14. Equilibrium configuration of geometry optimization for $[Fe^{III}(hfac)_3][Mn^{II}(acac)_2]$ system at the PBE0/def2-TZVP level of theory.

Table S9. Cartesian Coordinates for $[Fe^{III}(acac)_3][Mn^{II}(hfac)_2]$ (**4**) Optimized at the PBE0/def2-TZVP Level of Theory

Fe	3.586423000	3.012823000	10.644144000
Mn	1.825871000	5.707634000	11.777112000
O	2.821956000	2.026553000	12.198792000
O	5.311292000	2.149548000	11.103512000
O	4.545201000	4.027104000	9.230119000
O	3.890650000	4.674638000	11.796859000
O	3.005449000	1.677874000	9.325002000
O	1.697124000	3.873655000	10.438266000
O	0.609609000	4.626317000	13.160138000
O	2.218841000	6.924314000	13.498614000
O	0.155485000	6.917150000	11.163759000
O	2.870423000	7.042420000	10.476800000
C	2.484239000	0.754743000	14.137271000
H	2.014249000	1.591467000	14.658518000
H	3.019808000	0.130440000	14.850506000
H	1.677544000	0.177765000	13.678674000
C	3.377553000	1.288074000	13.062073000
C	4.736356000	0.954250000	13.053339000
H	5.111449000	0.308824000	13.834472000
C	5.623495000	1.389175000	12.070683000
C	7.055841000	0.953642000	12.109559000
H	7.293180000	0.425488000	11.182896000
H	7.271813000	0.309146000	12.959961000
H	7.698218000	1.836734000	12.147916000
C	6.326183000	5.158880000	8.211947000
H	6.607749000	4.243361000	7.688410000
H	7.214065000	5.738187000	8.458997000
H	5.698679000	5.743243000	7.533499000
C	5.516163000	4.810489000	9.419464000
C	5.837213000	5.378871000	10.662885000
H	6.719131000	5.999856000	10.725377000
C	4.999161000	5.338713000	11.763619000
C	5.335523000	6.119360000	12.987992000
H	4.576774000	6.889268000	13.149133000
H	6.315534000	6.587122000	12.913214000
H	5.305639000	5.459735000	13.858726000
C	1.769205000	0.218915000	7.975397000
H	2.387391000	0.402443000	7.092886000
H	0.748846000	0.004271000	7.662943000
H	2.190033000	-0.649998000	8.486016000
C	1.854612000	1.410539000	8.877619000
C	0.702400000	2.148340000	9.164472000
H	-0.231334000	1.798862000	8.748658000
C	0.663046000	3.316877000	9.918902000
C	-0.650856000	3.981537000	10.149322000
H	-0.863268000	3.976734000	11.222206000
H	-1.456631000	3.480422000	9.617125000
H	-0.607050000	5.029120000	9.846169000
C	0.225046000	4.972648000	14.292984000
C	0.640498000	6.065523000	15.053168000
H	0.202751000	6.228485000	16.024284000
C	1.616556000	6.947115000	14.589047000
C	0.152784000	8.022959000	10.589114000
C	1.228420000	8.692238000	10.005223000
H	1.067397000	9.652339000	9.543204000
C	2.504388000	8.130285000	9.994070000
C	3.639996000	8.910504000	9.304161000

F	4.614061000	9.174778000	10.177260000
F	4.167545000	8.178565000	8.316779000
F	3.241804000	10.069090000	8.777539000
F	-1.798365000	3.773790000	14.039800000
F	-0.244961000	2.853438000	15.219875000
F	-1.377163000	4.502736000	16.022260000
C	-0.820866000	4.023201000	14.909514000
F	1.377368000	8.142063000	16.661135000
F	1.876329000	9.273755000	14.896790000
F	3.349374000	7.985373000	15.804850000
C	2.050316000	8.103997000	15.509678000
F	-1.752684000	8.812839000	11.740917000
F	-1.224251000	9.885517000	9.948786000
F	-2.068090000	7.906680000	9.808438000
C	-1.239682000	8.679205000	10.519292000

Table S10. Cartesian Coordinates for [Mn^{III}(acac)$_3$][Fe^{II}(hfac)$_2$] (**4´**) Optimized at the PBE0/def2-TZVP Level of Theory

Mn	3.651225000	3.108623000	10.707557000
Fe	1.797296000	5.737787000	11.801070000
O	2.844444000	2.111289000	12.102406000
O	5.486272000	2.218488000	11.203751000
O	4.531062000	4.067573000	9.315345000
O	3.935831000	4.664361000	11.903273000
O	3.095980000	1.790837000	9.397239000
O	1.662358000	4.049496000	10.445005000
O	0.750912000	4.625017000	13.173354000
O	2.185783000	6.999816000	13.412792000
O	0.146623000	6.897352000	11.277514000
O	2.811321000	6.911888000	10.466982000
C	2.357038000	0.646204000	13.847267000
H	1.911403000	1.440958000	14.448860000
H	2.812266000	-0.097081000	14.499278000
H	1.544846000	0.188087000	13.277970000
C	3.346479000	1.243680000	12.897524000
C	4.672958000	0.845038000	12.936301000
H	4.949284000	0.098549000	13.667071000
C	5.677817000	1.350848000	12.088582000
C	7.078744000	0.829729000	12.223648000
H	7.337050000	0.280117000	11.314566000
H	7.204512000	0.174826000	13.084444000
H	7.769089000	1.672493000	12.295938000
C	6.255133000	5.241008000	8.270647000
H	6.602583000	4.326500000	7.785388000
H	7.099407000	5.892215000	8.487559000
H	5.581119000	5.745014000	7.574091000
C	5.489054000	4.879197000	9.500533000
C	5.826612000	5.447895000	10.732133000
H	6.687499000	6.098570000	10.773084000
C	5.006679000	5.379599000	11.847622000
C	5.316290000	6.190149000	13.057661000
H	4.533007000	6.938940000	13.199725000
H	6.281474000	6.686149000	12.976150000
H	5.302221000	5.543616000	13.938141000
C	1.861344000	0.293803000	8.106208000
H	2.529041000	0.421936000	7.250959000

H	0.852230000	0.091554000	7.752046000
H	2.229302000	-0.563975000	8.674224000
C	1.934123000	1.519435000	8.966520000
C	0.768790000	2.242988000	9.220907000
H	-0.146709000	1.847635000	8.804746000
C	0.664881000	3.437421000	9.942517000
C	-0.688622000	4.039738000	10.129785000
H	-0.891922000	4.129336000	11.199535000
H	-1.468500000	3.445104000	9.658166000
H	-0.705372000	5.052042000	9.722136000
C	0.336721000	4.982346000	14.297390000
C	0.691479000	6.113784000	15.025391000
H	0.255330000	6.278900000	15.996941000
C	1.617817000	7.027302000	14.521515000
C	0.132652000	7.975240000	10.656067000
C	1.195372000	8.585607000	9.983879000
H	1.037674000	9.525856000	9.481836000
C	2.449770000	7.989724000	9.944269000
C	3.570839000	8.696183000	9.163703000
F	4.606937000	8.949446000	9.965896000
F	4.008689000	7.909999000	8.173217000
F	3.186603000	9.850744000	8.619940000
F	-1.607728000	3.658822000	14.059955000
F	-0.026133000	2.874395000	15.298798000
F	-1.260230000	4.488171000	16.017204000
C	-0.660455000	3.996584000	14.932649000
F	1.391722000	8.252582000	16.571859000
F	1.790212000	9.368830000	14.771933000
F	3.342430000	8.161429000	15.658128000
C	2.030774000	8.220086000	15.401488000
F	-1.681480000	8.862205000	11.879597000
F	-1.226264000	9.846824000	10.017771000
F	-2.130863000	7.889924000	10.006735000
C	-1.243269000	8.665218000	10.637753000

Table S11. Cartesian Coordinates of Geometry Optimization for [Mn^{III}(hfac)$_3$][Fe^{II}(acac)$_2$] System at the PBE0/def2-TZVP Level of Theory

Mn	3.819335098	2.732963747	10.846889950
Fe	0.934426986	7.571290830	12.615716796
O	3.041933891	1.813387983	12.350073727
O	5.668166750	1.742190599	11.338639868
O	4.670051276	3.634415313	9.356094966
O	4.295225946	4.208287526	11.999659970
O	3.322392478	1.285741549	9.702477861
O	1.952324165	3.665132202	10.314631100
O	0.433678824	5.828957906	13.354511378
O	1.447652129	8.217042009	14.400010295
O	-0.439488654	8.578245118	11.632603435
O	2.208587646	7.781147426	11.124331989
C	3.519657729	0.869487524	13.046825050
C	4.786046141	0.328702174	13.021376508
H	5.021900764	-0.478979405	13.694491107
C	5.784592325	0.829939416	12.167035200
C	5.283780172	4.731383363	9.376531709
C	5.475704115	5.571993122	10.461054063
H	6.017643341	6.494812861	10.339495973

C	4.948742441	5.237835048	11.701582450
C	2.246702552	1.092934610	9.054832272
C	1.147773819	1.909009478	8.945275134
H	0.312755420	1.586182814	8.343550419
C	1.094506160	3.167959472	9.579341566
C	0.466309791	5.468919253	14.569622166
C	0.910377377	6.269355120	15.628110713
H	0.892254138	5.841729613	16.620801627
C	1.372350962	7.583368230	15.494997986
C	-0.329051243	9.116617478	10.493636710
C	0.827615586	9.080187917	9.703324118
H	0.798535111	9.589518812	8.750253964
C	2.011958535	8.424120384	10.046699753
C	2.257135465	-0.255342977	8.319166052
F	3.276525941	-0.302940922	7.466174666
F	2.399380903	-1.248383949	9.195632506
F	1.134663712	-0.466816926	7.635119404
C	-0.147950262	4.036681299	9.281046858
F	-0.054463848	4.522723953	8.036904145
F	-1.266128056	3.310013132	9.350618965
F	-0.249573931	5.048769089	10.119333604
C	2.496731725	0.317889149	14.053041082
F	2.192775895	1.257679991	14.948604698
F	1.378329736	-0.034285683	13.424683729
F	2.953605810	-0.744465340	14.711413076
C	7.198356930	0.216424771	12.246358222
F	8.081776130	1.162561322	12.557529680
F	7.287406368	-0.748493664	13.161998608
F	7.534303750	-0.300351335	11.068069368
C	5.837939703	5.135086274	7.999606375
F	6.559473210	6.251899515	8.060517244
F	6.603726502	4.168841172	7.511370364
F	4.826066305	5.340644730	7.157643645
C	5.203106472	6.167877800	12.901136568
F	5.655354618	7.357858671	12.517897156
F	4.098366625	6.339175266	13.607351715
F	6.125477962	5.607612572	13.687133708
C	-0.018859276	4.078062666	14.846018867
H	-1.059123072	3.992777983	14.521712510
H	0.562786326	3.370619162	14.251139353
H	0.054292134	3.810900767	15.899077601
C	1.819016634	8.335754247	16.713361689
H	1.735838723	7.743905567	17.623698037
H	2.856049204	8.652860379	16.579330891
H	1.217125298	9.242062253	16.814850073
C	-1.548562524	9.825662830	9.984337853
H	-1.368183053	10.346703901	9.045392156
H	-2.348457416	9.094671426	9.840049280
H	-1.895327963	10.535800866	10.737910789
C	3.160429105	8.446262692	9.080846974
H	4.074294555	8.727443619	9.608218925
H	3.306705147	7.440590778	8.676842675
H	2.992655307	9.131439681	8.251278576

Table S12. Cartesian Coordinates of Geometry Optimization for $[Fe^{III}(hfac)_3][Mn^{II}(acac)_2]$ System at the PBE0/def2-TZVP Level of Theory

----

Fe	3.749564396	2.654238890	10.924635814
Mn	0.937321000	7.601603607	12.530020975
O	3.022994247	1.674162443	12.521891620
O	5.467677582	1.680572647	11.301936568
O	4.645438522	3.629656193	9.422965422
O	4.246226139	4.227206330	12.058591626
O	3.215718221	1.205564702	9.643315269
O	1.948028781	3.442842172	10.559846776
O	0.447030169	5.782277474	13.271092487
O	1.355959924	8.243519633	14.410851416
O	-0.432454316	8.659508980	11.460264171
O	2.282366276	7.834611415	11.012117118
C	3.553477636	0.776595433	13.216799125
C	4.844558289	0.274965713	13.102127759
H	5.179586010	-0.500840168	13.770509406
C	5.713123114	0.787612033	12.144441473
C	5.285105167	4.708666153	9.444248973
C	5.479124204	5.549835805	10.530329979
H	6.042474208	6.460149584	10.410851894
C	4.921833269	5.240242262	11.766878154
C	2.186651606	1.110248098	8.933500590
C	1.099897133	1.975076425	8.908176217
H	0.272316281	1.778154048	8.246698395
C	1.064194196	3.083064321	9.750362509
C	0.470322606	5.423611840	14.484796936
C	0.846418397	6.234172323	15.564374209
H	0.815913702	5.789416198	16.549426953
C	1.261563060	7.569415394	15.477939872
C	-0.262930257	9.195046901	10.328665335
C	0.918697359	9.144393321	9.574497855
H	0.917145907	9.656142787	8.622160602
C	2.097055716	8.483414447	9.939255624
C	2.183430812	-0.128544516	8.020806178
F	3.225247037	-0.088430905	7.196229276
F	2.274142113	-1.232422158	8.759273961
F	1.075148661	-0.211535537	7.286676336
C	-0.193909828	3.972437100	9.753607532
F	0.140402830	5.254823014	9.791864445
F	-0.943512932	3.770494638	8.668340877
F	-0.933290942	3.689815994	10.822978920
C	2.627713966	0.217824948	14.312090006
F	2.339423215	1.177039196	15.189837532
F	1.490768208	-0.211257036	13.772281842
F	3.181725262	-0.795762381	14.974112536
C	7.152505898	0.247455572	12.068562963
F	8.016318393	1.240458965	12.265887219
F	7.386615012	-0.693114350	12.982244225
F	7.379139231	-0.274825311	10.867685823
C	5.873244178	5.099189659	8.077652234
F	6.592489902	6.218837423	8.137858552
F	6.653529172	4.128215932	7.617884113
F	4.883737398	5.291953240	7.204467019
C	5.161669802	6.190489340	12.953298435
F	5.719982576	7.337476051	12.575206395
F	4.022605557	6.460698746	13.574733806
F	5.983814456	5.599402912	13.823918420
C	0.054783889	4.006318714	14.747741410
H	-0.955851118	3.854563483	14.360780392
H	0.714256364	3.335779139	14.191849796
H	0.082658140	3.746811256	15.804862022
C	1.637966522	8.295466273	16.736735086
H	1.507771020	7.686480068	17.629960993
H	2.680961221	8.613851416	16.663832044

```
H        1.032632015      9.200997136     16.820365207
C       -1.445138784      9.937576489      9.776846400
H       -2.287918490      9.247581833      9.689671234
H       -1.741022782     10.713190124     10.487203738
H       -1.245511579     10.389936502      8.806739259
C        3.259824796      8.515732949      8.988994280
H        4.162455638      8.802890412      9.532203028
H        3.422339975      7.509470169      8.593271823
H        3.101963217      9.197542309      8.154771027
----------------------------------------------------------------------------
```

Table S13. Absolute Energies for Heteroleptic Fe-Mn Systems Calculated at Different Levels of Theory

System	PBE0/def2-TZVP	B2PLYP-D3/TZVP/ZORA
[Fe^{III}(acac)$_3$][Mn^{II}(hfac)$_2$] (**4**)	-5329.6559391	-5359.771578668012
[Mn^{III}(acac)$_3$][Fe^{II}(hfac)$_2$] (**4´**)	-5329.6423313	-5359.755994653803
[Mn^{III}(hfac)$_3$][Fe^{II}(acac)$_2$]	-5924.7851120	-
[Fe^{III}(hfac)$_3$][Mn^{II}(acac)$_2$]	-5924.7946172	-

ATR-IR Spectra of Diketonate Precursors

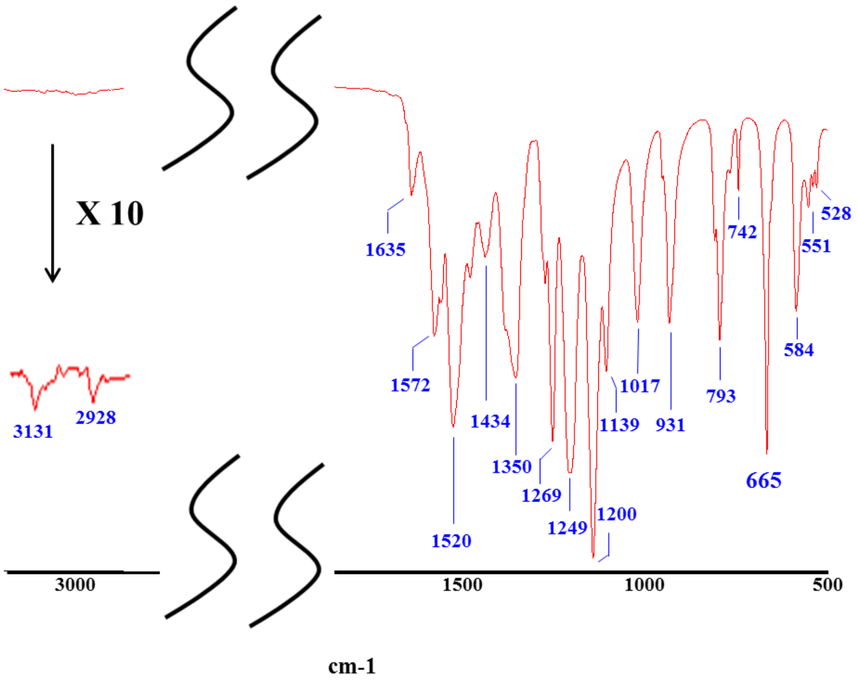

Figure S15. The attenuated total reflection (ATR) spectrum of [Fe(acac)$_3$][Fe(hfac)$_2$] (**1**).

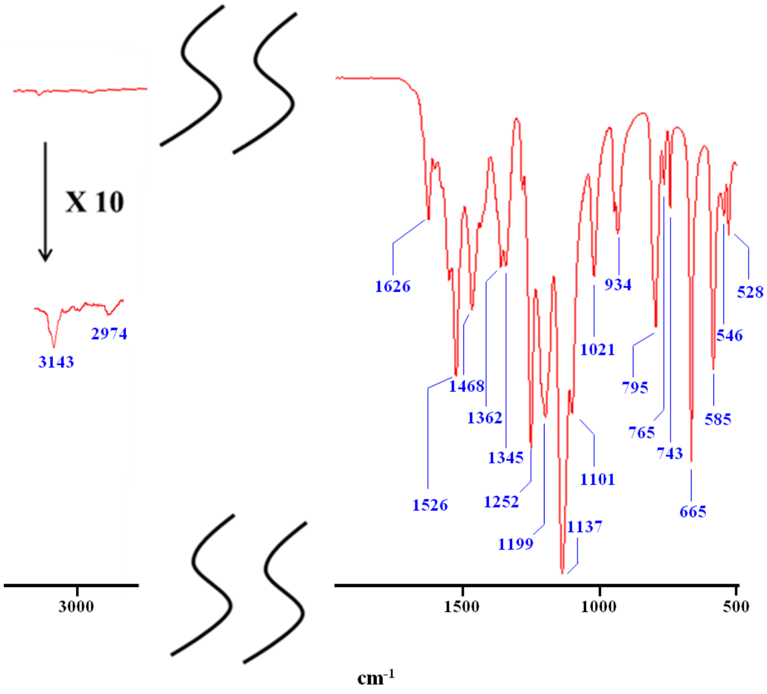

Figure S16. The attenuated total reflection (ATR) spectrum of [Fe(hfac)$_2$][Fe(acac)$_3$][Fe(hfac)$_2$] (**2**).

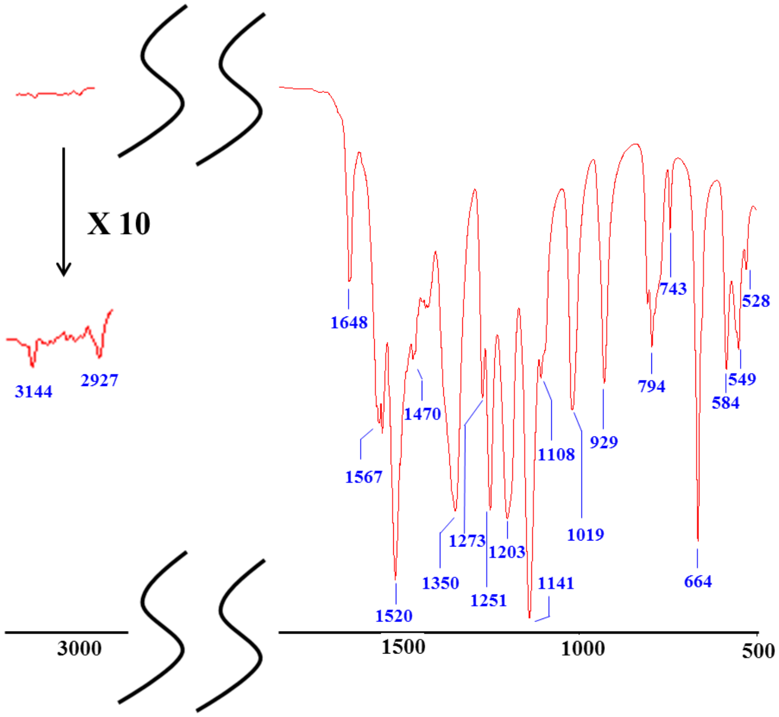

Figure S17. The attenuated total reflection (ATR) spectrum of $[Fe(acac)_3][Mn(hfac)_2]$ (**4**).

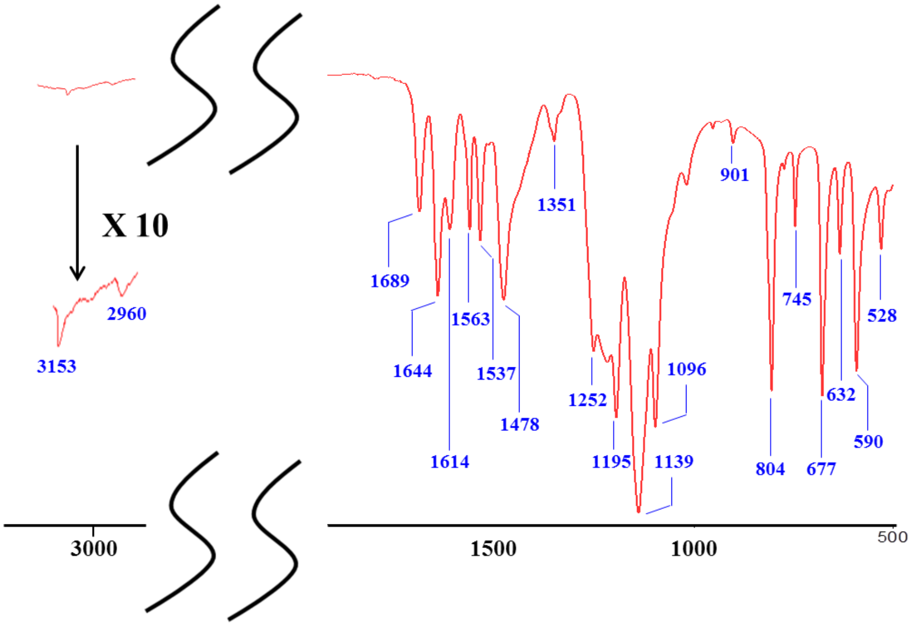

Figure S18. The attenuated total reflection (ATR) spectrum of $[Ni(hfac)_2][Fe(acac)_3][Ni(hfac)_2]$ (**5**).

Analysis of Decomposition Residues of Diketonate Precursors

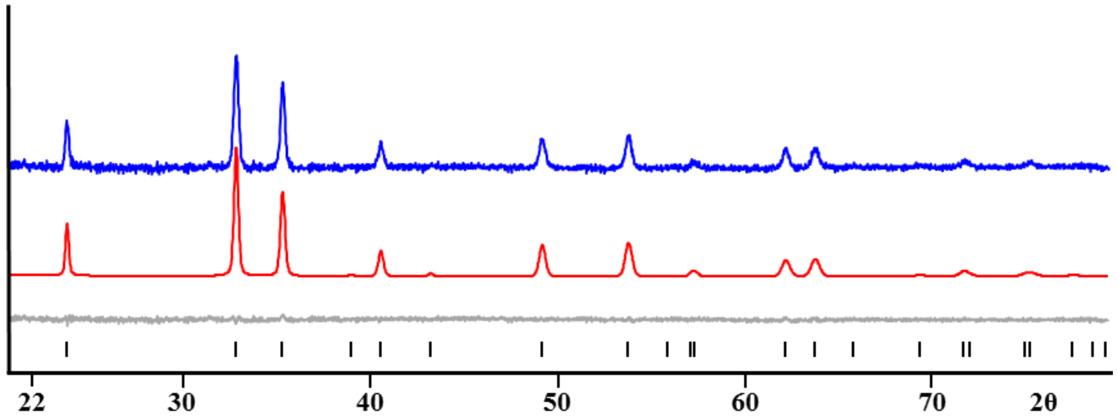

Figure S19. X-ray powder diffraction pattern of oxide sample obtained by decomposition of homometallic complex [Fe(acac)$_3$][Fe(hfac)$_2$] (**1**) in air at 500 ^{o}C and the Le Bail fit for the trigonal unit cell (Sp. gr. *R*-3*c*). Blue and red curves are experimental and calculated patterns. Grey line is the difference curve. Theoretical peak positions for hematite Fe_2O_3 (PDF No. 00-033-0664) are shown at the bottom.

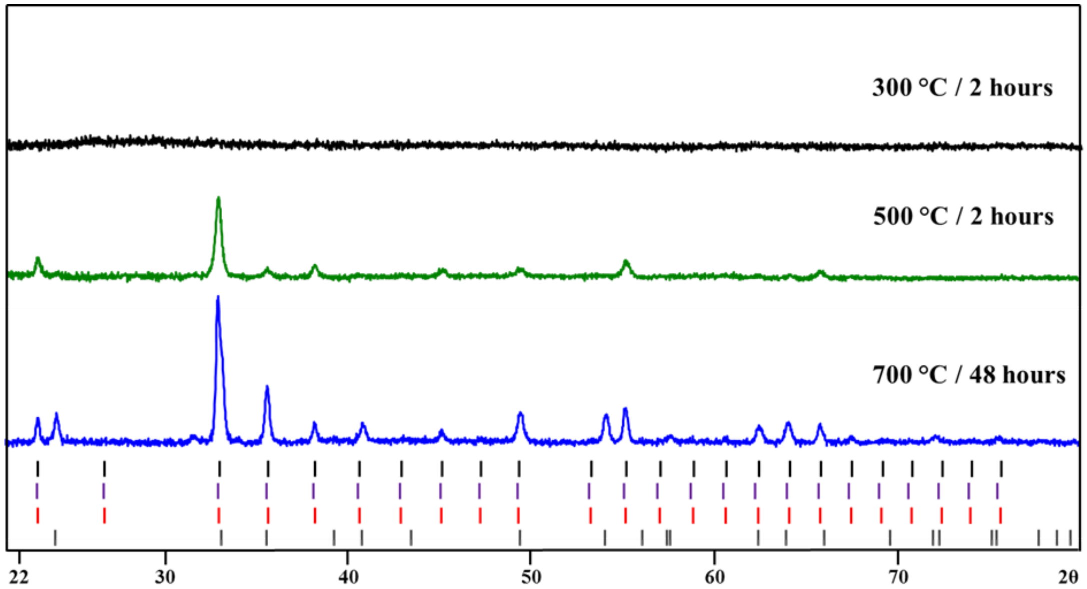

Figure S20. X-ray powder diffraction patterns of the residues obtained by decomposition of heterometallic precursor [Fe(acac)$_3$][Mn(hfac)$_2$] (**4**) in air at 300 °C (black), 500°C (green), and 700°C (blue). Theoretical peak positions for Fe_2O_3 (Sp. gr. *Ia*-3),[3] Mn_2O_3 (*Ia*-3),[4] $FeMnO_3$ (*Ia*-3),[5] and Fe_2O_3 (*R*-3*c*)[6] are shown at the bottom as black, purple, red, and grey lines, respectively.

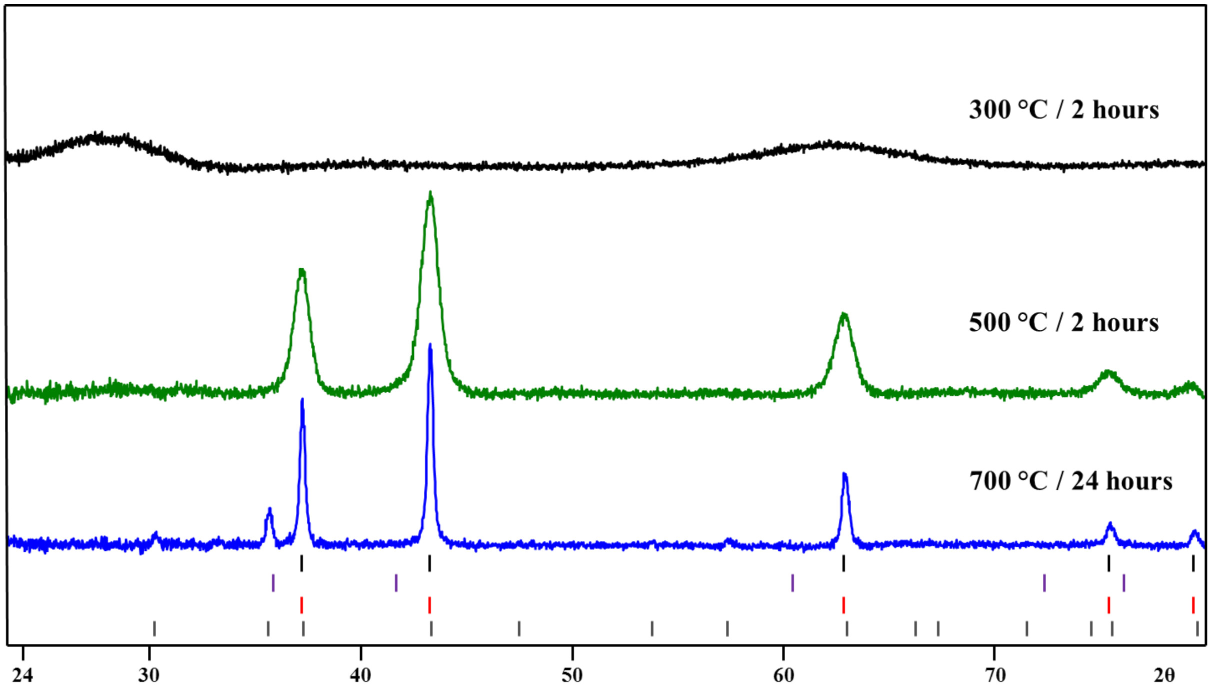

Figure S21. X-ray powder diffraction patterns of the residues obtained by decomposition of heterometallic precursor [Ni(hfac)$_2$][Fe(acac)$_3$][Ni(hfac)$_2$] (**5**) in air at 300 °C (black), 500 °C (green), and 700 °C (blue). Theoretical peak positions for NiO (Sp. gr. *Fm*-3*m*),[7] FeO (*Fm*-3*m*),[8] $Fe_{0.05}Ni_{0.95}O$ (*Fm*-3*m*),[9] and $Fe_{2.1}Ni_{0.9}O_4$ (*Fd*-3*m*)[9] are shown at the bottom as black, purple, red, and grey lines, respectively.

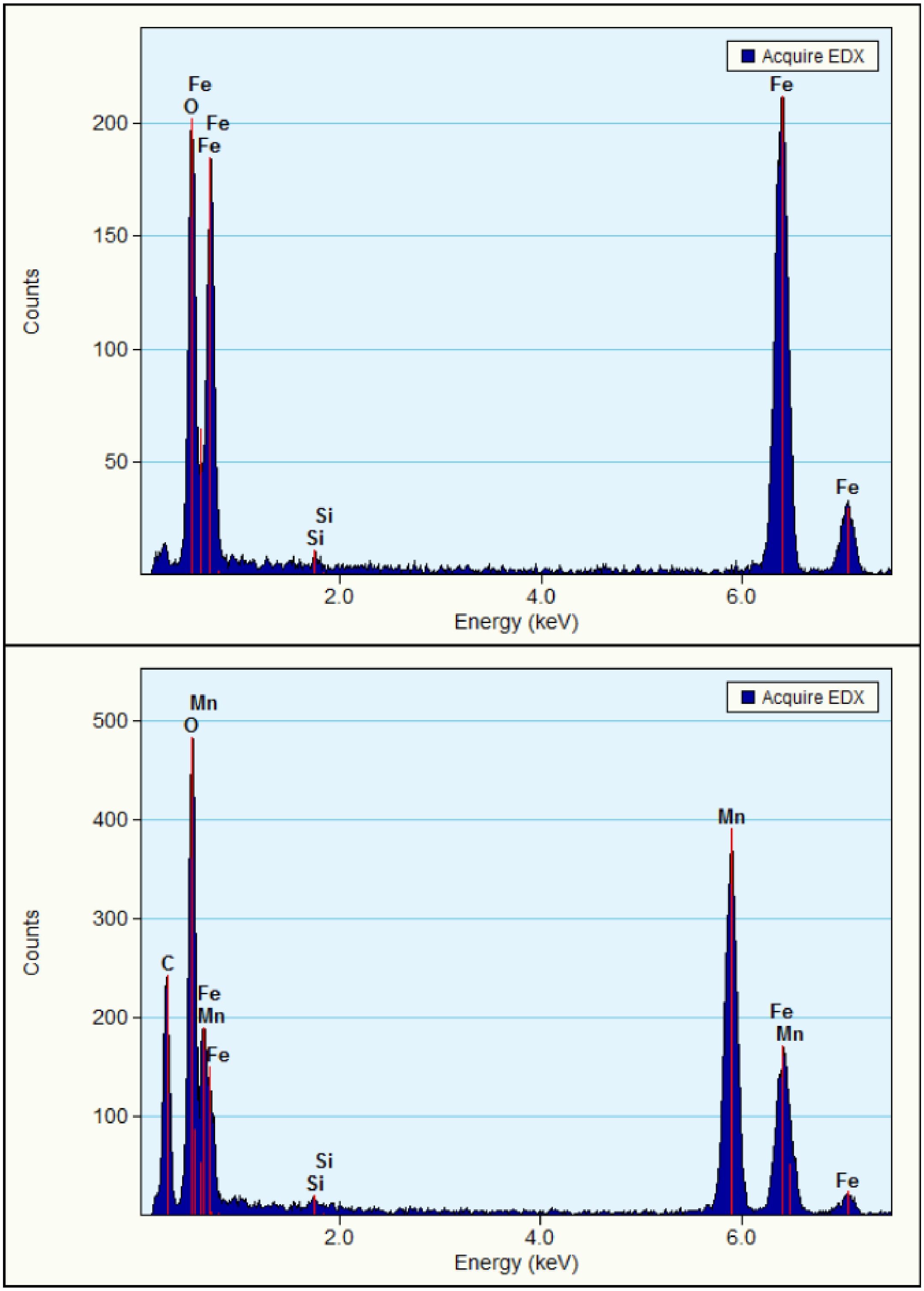

Figure S22. Energy dispersive X-ray (EDX) spectra for the two phases in the sample obtained by decomposition of heterometallic precursor [Fe(acac)$_3$][Mn(hfac)$_2$] (**4**) in air at 500°C: Fe_2O_3 oxide (top) and $Fe_xMn_{2-x}O_3$ oxide (bottom).

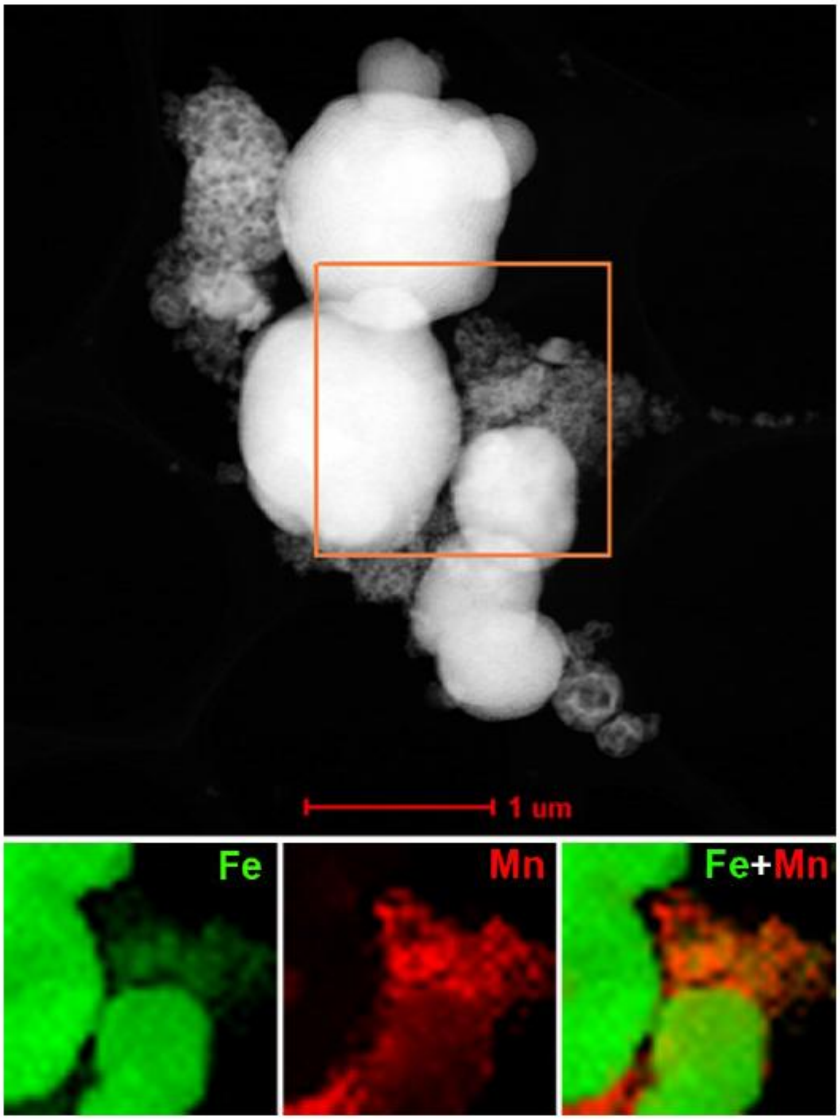

Figure S23. EDX Fe-Mn map of the sample obtained by decomposition of heterometallic precursor [Fe(acac)$_3$][Mn(hfac)$_2$] (**4**) in air at 500°C showing two phases: large micron-size particles of Fe_2O_3 oxide and agglomerated nanocrystals of $Fe_xMn_{2-x}O_3$ oxide.

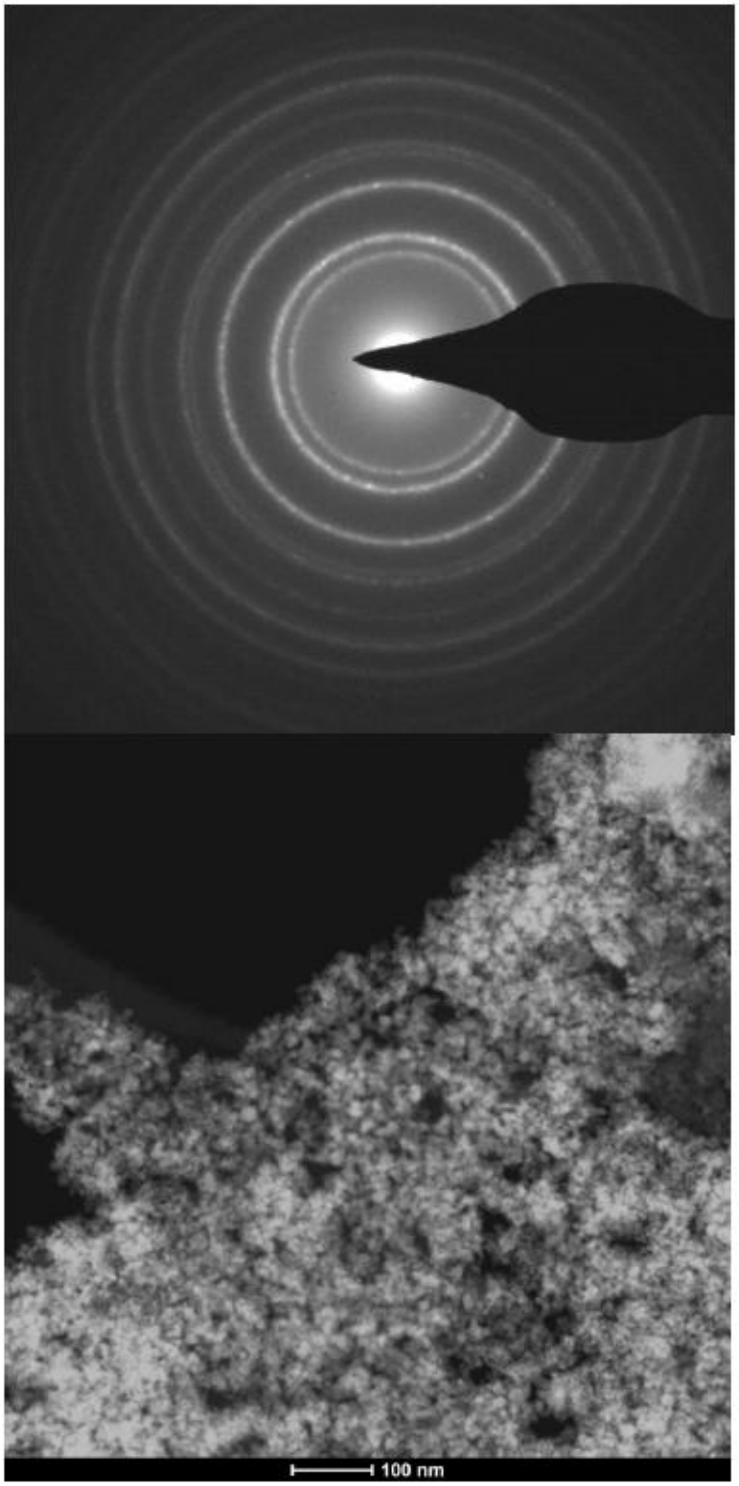

Figure S24. Electron diffraction pattern and overview high angle annular dark field scanning transmission electron microscopy (HAADF-STEM) image of the sample obtained by decomposition of heterometallic precursor [Ni(hfac)$_2$][Fe(acac)$_3$][Ni(hfac)$_2$] (**5**) in air at 500°C. The electron diffraction pattern corresponds to the face-centered cubic structure with $a \approx 4.3$ Å.

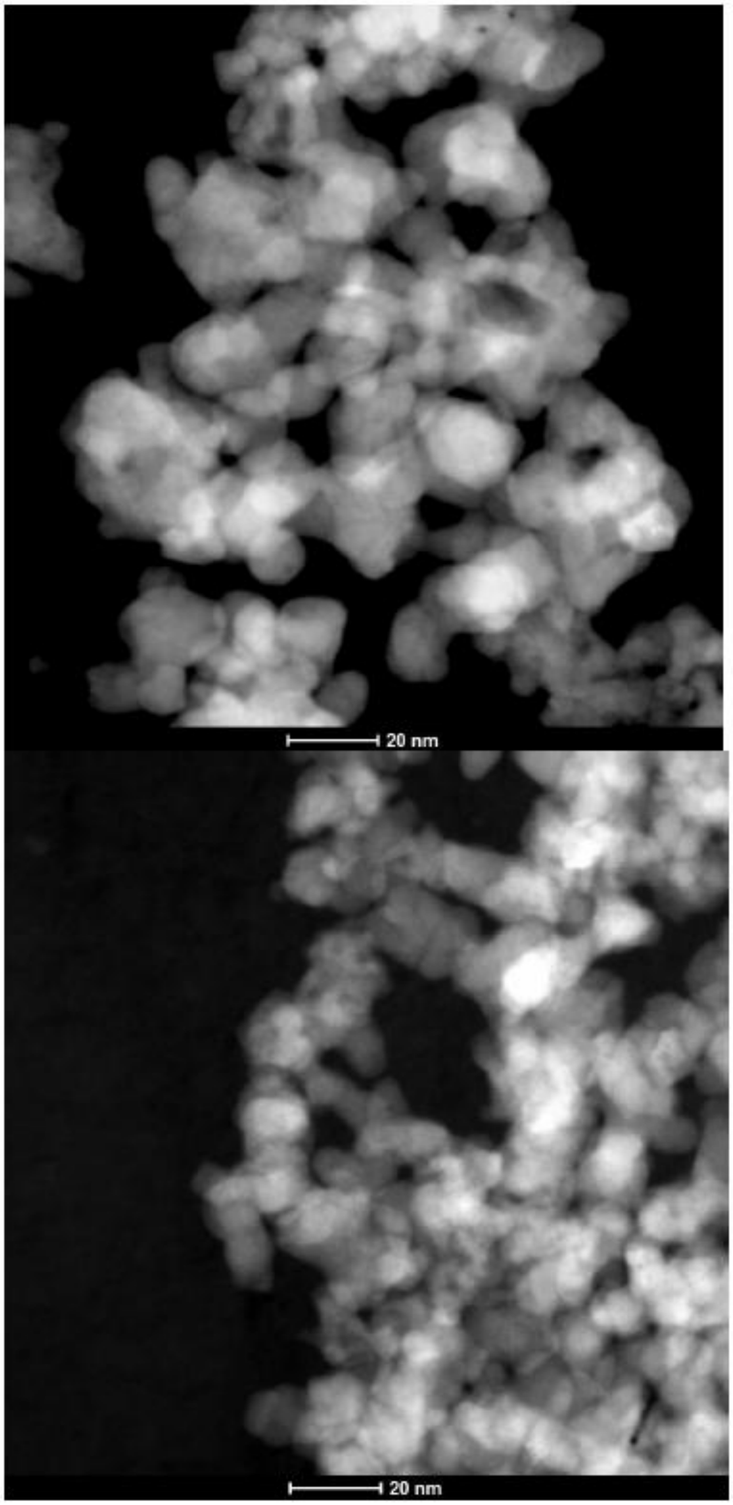

Figure S25. HAADF-STEM image of the sample obtained by decomposition of heterometallic precursor [Ni(hfac)$_2$][Fe(acac)$_3$][Ni(hfac)$_2$] (**5**) in air at 500°C showing agglomerated nanoparticles.

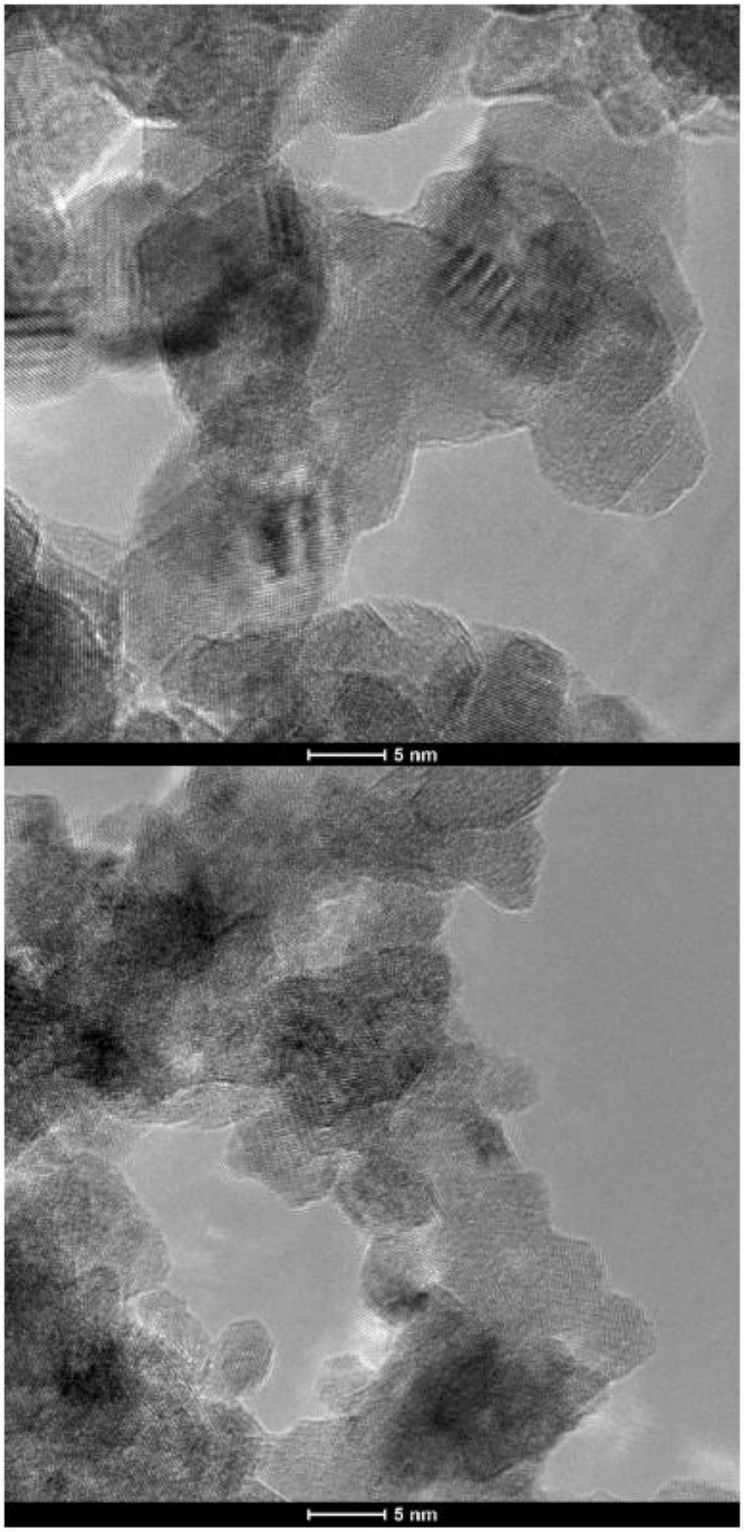

Figure S26. Bright field (BF TEM) image of the sample obtained by decomposition of heterometallic precursor [Ni(hfac)$_2$][Fe(acac)$_3$][Ni(hfac)$_2$] (**5**) in air at 500°C showing crystalline agglomerated nanoparticles.

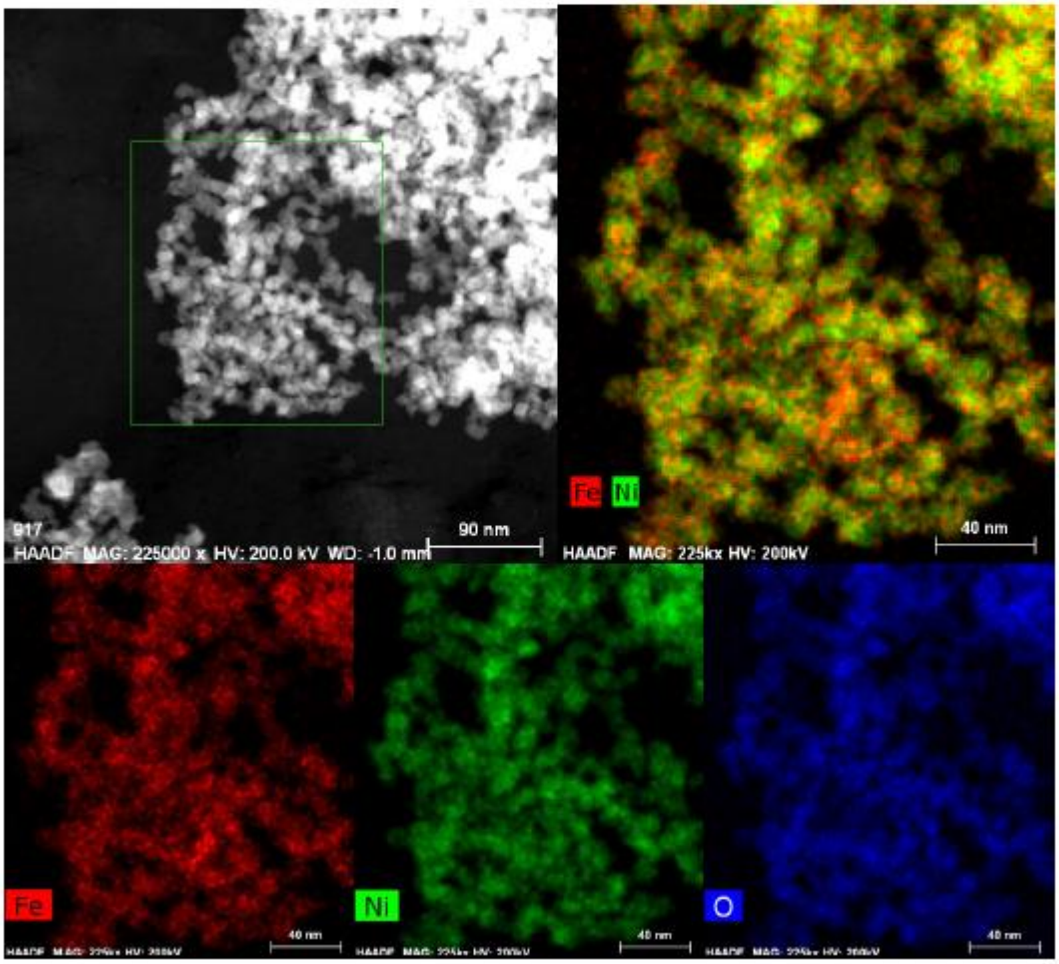

Figure S27. EDX Fe-Ni-O map of the sample obtained by decomposition of heterometallic precursor [Ni(hfac)$_2$][Fe(acac)$_3$][Ni(hfac)$_2$] (**5**) in air at 500°C.

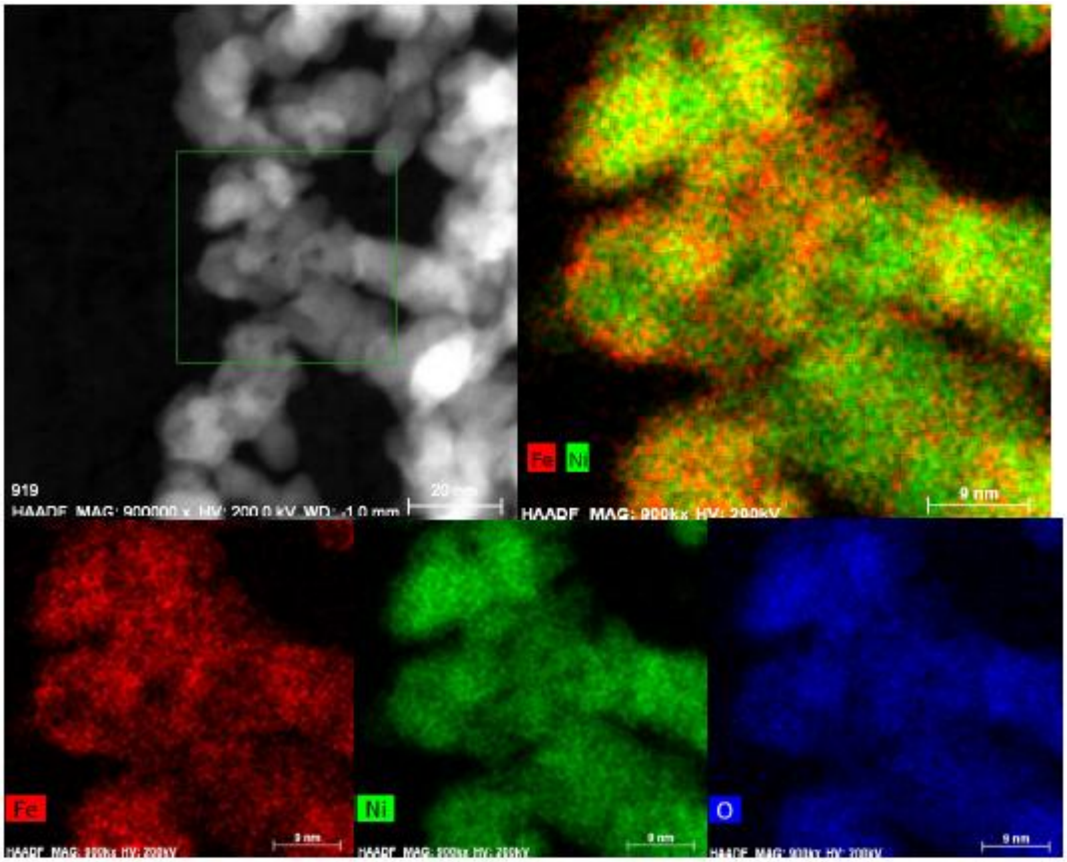

Figure S28. EDX Fe-Ni-O map of the sample obtained by decomposition of heterometallic precursor [Ni(hfac)$_2$][Fe(acac)$_3$][Ni(hfac)$_2$] (**5**) in air at 500°C.

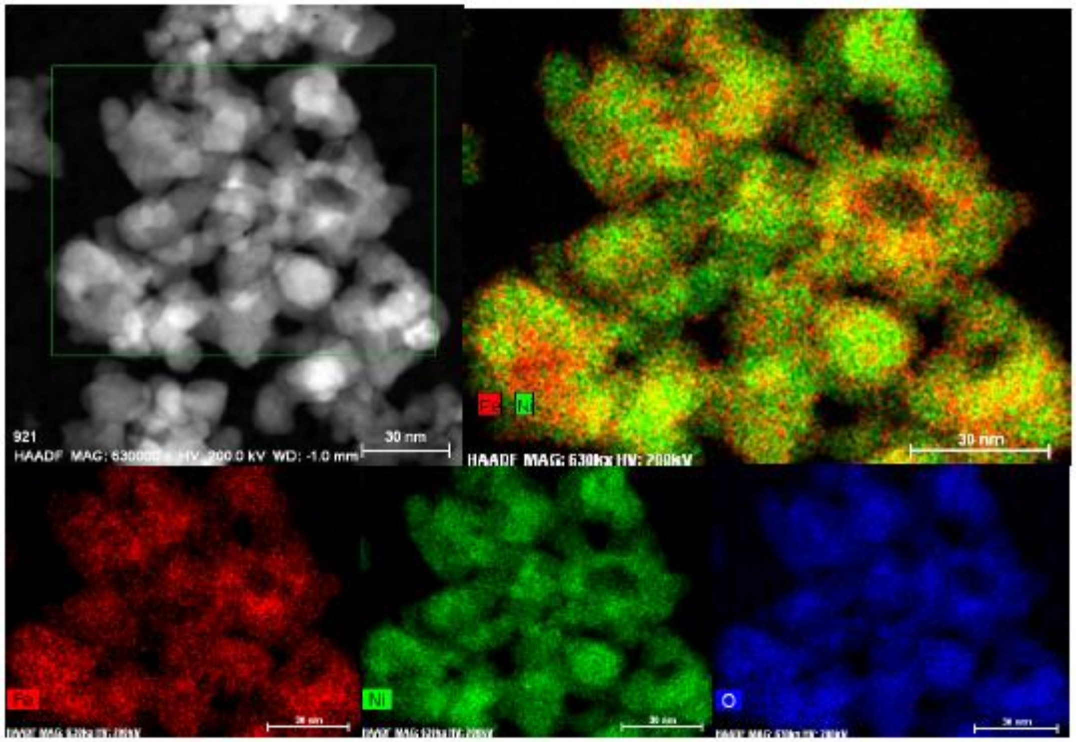

Figure S29. EDX Fe-Ni-O map of the sample obtained by decomposition of heterometallic precursor [Ni(hfac)$_2$][Fe(acac)$_3$][Ni(hfac)$_2$] (**5**) in air at 500°C.

References

[1] (a) C. E. Pfluger, and P. S. Haradem, *Inorg. Chim. Acta*, 1983, **69**, 141; (b) J. R. Bryant, J. E. Taves, and J. M. Mayer, *Inorg. Chem.* 2002, **41**, 2769.

[2] H. Zhang, B. Li, J. Sun, R. Clérac, and E. V. Dikarev, *Inorg. Chem.*, 2008, **47**, 10046.

[3] L. Ben-Dor, E. Fischbein, , Z. H. Kalman, *Acta Crystallogr., Sec. B,* 1976, **32**, 667.

[4] S. Geller, *Acta Crystallogr., Sec. B*, 1971, **27**, 821.

[5] H. Dachs, *Z. Kristallogr., Kristallgeom., Kristallphys., Kristallchem.*, 1956, **107**, 370.

[6] Natl. Bur. Stand. (U.S.) Monogr. 25, 1981, **18**, 37.

[7] S. Sasaaki, K. Fujino, and Y. Takeuchi, *Proc. JPN. Acad., Ser. B*, 1979, **55**, 43.

[8] H. Fjellvag, F. Gronvold, S. Stolen, and B. Hauback, *J. Solid State Chem.*, 1996, **124**, 52.

[9] E. A. Kiselev, N. V. Proskurnina, V. I. Voronin, V. A. Cherepanov, *Neorg. Mater.*, 2007, **43**, 209.
